# Cell-type specific effects of mineralocorticoid receptor gene expression suggest intercellular communication regulating fibrosis in skeletal muscle disease

**DOI:** 10.3389/fphys.2024.1322729

**Published:** 2024-04-26

**Authors:** Chetan K. Gomatam, Pratham Ingale, Gabriel Rodriguez, Sarah Munger, Rachel Pomeranets, Swathy Krishna, Jeovanna Lowe, Zachary M. Howard, Jill A. Rafael-Fortney

**Affiliations:** Department of Physiology and Cell Biology, College of Medicine, The Ohio State University, Columbus, OH, United States

**Keywords:** Duchenne muscular dystrophy, fibrosis, lysyl oxidase, mineralocorticoid receptor, gene expression

## Abstract

**Introduction:** Duchenne muscular dystrophy (DMD) is a fatal striated muscle degenerative disease. DMD is caused by loss of dystrophin protein, which results in sarcolemmal instability and cycles of myofiber degeneration and regeneration. Pathology is exacerbated by overactivation of infiltrating immune cells and fibroblasts, which leads to chronic inflammation and fibrosis. Mineralocorticoid receptors (MR), a type of nuclear steroid hormone receptors, are potential therapeutic targets for DMD. MR antagonists show clinical efficacy on DMD cardiomyopathy and preclinical efficacy on skeletal muscle in DMD models.

**Methods:** We have previously generated myofiber and myeloid MR knockout mouse models to dissect cell-specific functions of MR within dystrophic muscles. Here, we compared skeletal muscle gene expression from both knockouts to further define cell-type specific signaling downstream from MR.

**Results:** Myeloid MR knockout increased proinflammatory and profibrotic signaling, including numerous myofibroblast signature genes. *Tenascin C* was the most highly upregulated fibrotic gene in myeloid MR-knockout skeletal muscle and is a component of fibrosis in dystrophic skeletal muscle. Surprisingly, *lysyl oxidase* (*Lox*), canonically a collagen crosslinker, was increased in both MR knockouts, but did not localize to fibrotic regions of skeletal muscle. Lox localized within myofibers, including only a region of quadriceps muscles. *Lysyl oxidase like 1* (*Loxl1*), another Lox family member, was increased only in myeloid MR knockout muscle and localized specifically to fibrotic regions.

**Discussion:** This study suggests that MR signaling in the dystrophic muscle microenvironment involves communication between contributing cell types and modulates inflammatory and fibrotic pathways in muscle disease.

## Introduction

Duchenne muscular dystrophy (DMD) is an X-linked genetic disease of striated muscles that affects 1:5,000 boys (Mendell et al., 2012). It is caused by mutations in the *DMD* gene, which encodes the dystrophin protein. Dystrophin is crucial for maintaining stability of striated muscle membranes during contraction due to its role in linking the cytoskeleton with the extracellular matrix through binding the dystrophin-glycoprotein complex (Ahn and Kunkel, 1993). The absence of dystrophin leads to increased susceptibility of the sarcolemma to contraction-induced membrane tears causing repetitive injury of the muscle to occur. The resultant cycles of degeneration and regeneration in skeletal muscle lead to chronic muscle inflammation and eventually deleterious fibrosis replacing muscle tissue.

Extracellular matrix deposition is necessary for normal wound healing to temporarily hold injured tissue together and to act as a scaffold for satellite cells to regenerate new myofibers (Klingler et al., 2012; Bentzinger et al., 2013; Tidball, 2017). In DMD, continual cycles of degeneration and regeneration lead to the overactivation of infiltrating immune cells and fibroblasts, which are components of the normal injury response (Tidball, 2017; Tidball et al., 2018). Immune cells normally function to clear cellular debris from the injury site. Fibroblasts are largely responsible for producing extracellular matrix (Bentzinger et al., 2013; Tidball, 2017; Tidball et al., 2018). However, overactivation of both cell types results in persistent inflammation and fibrosis that eventually replaces muscle tissue (Bentzinger et al., 2013; Tidball, 2017).

We have previously demonstrated that the mineralocorticoid receptor (MR) is present in skeletal muscle and represents a therapeutic target for treatment of skeletal muscle pathology in DMD (Rafael-Fortney et al., 2011; Chadwick et al., 2015; Lowe et al., 2016; Lowe et al., 2020). MR is a nuclear steroid hormone receptor whose canonical functions include maintaining electrolyte balance in the kidneys and blood pressure to support normal heart function (Delyani et al., 2001; Tamargo et al., 2014). MR acts by binding to its ligand aldosterone, migrating into the nucleus, and promoting transcription of numerous genes. MR antagonists (MRAs) have a long history of safety and efficacy in heart disease and have clinically demonstrated efficacy for DMD cardiomyopathy (Messaoudi et al., 2012; Raman et al., 2015; Raman et al., 2019).

With the goal of translating MRA use for the earlier onset skeletal muscle pathology in DMD, we have been defining the mechanisms of MR function in mouse skeletal muscles using a combination of pharmacological and genetic approaches. We have shown that inflammatory myeloid cells in dystrophic muscles contain high levels of aldosterone synthase or CYP11B2, the enzyme required for synthesis of the endogenous MR agonist aldosterone (Chadwick et al., 2016). Increased muscle aldosterone levels in dystrophic muscles lead to overactive transcription of MR-regulated genes, which can be blocked by MRAs, providing overall rationale for MRA efficacy in dystrophic skeletal muscle (Chadwick et al., 2016; Chadwick et al., 2017). In dystrophin-deficient *mdx* mice, genetic deletion of myofiber MR improves muscle force and reduces fibrosis, but does not improve muscle membrane integrity (Hauck et al., 2019). However, MRAs directly stabilize dystrophic muscle membranes independent of myofiber MR (Chadwick et al., 2017; Hauck et al., 2019). Myeloid immune cells isolated from MRA-treated *mdx* mice show reduced expression of the gene encoding fibronectin, an extracellular matrix protein and fibrosis component known to augment fibroblast proliferation and activation in other tissues (Patten and Wang, 2021; Rudnik et al., 2021; Howard et al., 2022a). In contrast, isolated genetic deletion of myeloid MR in *mdx* mice increases muscle fibrosis (Howard et al., 2022b).

To further delineate the effects of the MR knockouts, we compared the gene expression of quadriceps muscles from *mdx* mice with muscles from littermates with knockout of MR in either myofibers or myeloid cells. We also investigated the protein-level expression of several key differentially expressed genes. These data reveal the cell specific roles of MR regulated gene expression within the complex dystrophic microenvironment.

## Materials and methods

### Animals

All mouse protocols were approved by the Institutional Animal Care and Use Committee of the Ohio State University, comply with all laws of the United States of America, and conform to the National Institutes of Health Guide for the Care and Use of Laboratory Animals. All mice used in experiments were euthanized by cervical dislocation to avoid chemical contamination of muscle tissues, as approved by the above guidelines. *Mdx* mice with a myeloid MR knockout (LysM-Cre; MR^flox/flox^; *mdx* or LysM-MRcko/*mdx*) or a myofiber MR knockout (MCK-Cre; MR^flox/flox^; *mdx* or MCK-MRcko/*mdx*) were bred and genotyped as previously described (Hauck et al., 2019; Howard et al., 2022b). Gene expression microarray was performed on quadriceps from 8-week-old LysM-MRcko/*mdx* [*n* = 3; 2 M, 1 F], MCK-MRcko/*mdx* [*n* = 3; 1 M, 2 F], and Cre-/*mdx* control mice [*n* = 3, 2 M, 1 F].

### Gene expression microarray

RNA was isolated from frozen quadriceps muscles after homogenization using TRIzol reagent (Life Technologies, Grand Island, NY, United States), according to the manufacturer’s instructions, followed by incubation with DNAse I (RQ1; Promega, Madison, WI, United States). DNAse-treated RNA concentration was determined spectrophotometrically, and samples were further purified using the RNeasy mini kit (Qiagen, Valencia, CA, United States) cleanup protocol, and final RNA concentrations were determined.

RNA integrity was measured using the Agilent 2100 Bioanalyzer (Agilent Technologies, Palo Alto, CA, United States) and all samples had RNA integrity numbers above 7. 100 ng of total mRNA was linearly amplified. Then 5.5 µg of cDNA was labeled and fragmented using the GeneChip WT Plus reagent kit (Affymetrix, Santa Clara, CA, United States) following the manufacturer’s instructions. Labeled cDNA targets were hybridized to Affymetrix GeneChip Clariom D array, Mouse for 16 h at 45°C rotating at 60 rpm. The arrays were washed and stained using the Fluidics Station 450 and scanned using the GeneChip Scanner 3000 7G. Arrays were normalized using the gene-level SST RMA algorithm in Expression Console, and comparisons were made using Transcriptome Analysis Console software (Affymetrix). The microarray data have been deposited in the NCBI Gene Expression Omnibus (accession number: GSE244569). Gene groups were determined using the functional annotation clustering tool from the Database for Annotation, Visualization, and Integrated Discovery (DAVID). Gene expression was further analyzed and gene ontology (GO) Plots were generated using the WEB-based GEne SeT AnaLysis Toolkit (WebGestalt) (Liao et al., 2019).

### Immunofluorescence staining

Muscles were isolated from mice and frozen in optimal cutting temperature media (OCT) on liquid nitrogen-cooled isopentane and 8-micron sections were cut onto microscope slides. Eight-week-old and one-year-old LysM-MRcko/*mdx* (1 yr: n = 6; 8 wk: *n* = 6) and Cre-/*mdx* (1 yr: *n* = 8; 8 wk: *n* = 6), and 8-week-old MCK-MRcko/*mdx* (8 wk: *n* = 6) quadriceps slides were incubated with 1:100 tenascin C primary antibody (Millipore, AB19011), followed by 1:200 Alexa Fluor 555 goat-anti-rabbit (Thermofisher, A21429) secondary antibody. Eight-week-old LysM-MRcko/*mdx* (*n* = 4), MCK-MRcko/*mdx* (*n* = 4), Cre-/*mdx* (*n* = 4), and C57 wild-type (*n* = 2) quadriceps were incubated with the following rabbit primary antibodies: 1:40 fibronectin (Abcam, ab23750), 1:100 Col I (Abcam, ab34710), 1:200 Lox (Abcam, ab174316) or 1:100 Lox [Invitrogen, MA5-32817 (not shown)], and 1:100 Loxl1 (Abnova, H00004016D01P) followed by 1:200 Alexa Fluor 555 goat anti-rabbit IgG (Thermofisher, A21429). Multiple muscles (heart, diaphragm, *tibialis anterior*, gastrocnemius, soleus, *extensor digitorum longus*, abdominals, *gluteus maximus*, and triceps) from one 9-week-old C57 wild-type mouse (M) were incubated with 1:300 Lox (Abcam, ab174316) or 1:100 Lox [Invitrogen, MA5-32817 (not shown)]. Longitudinal quadriceps sections and transverse heart sections from 7-8-week-old C57 wild-type (*n* = 2) and *mdx* (*n* = 2) mice were incubated with 1:100 Lox (Invitrogen, MA5-32817). LysM-MRcko/*mdx* (*n* = 5), MCK-MRcko/*mdx* (*n* = 6), Cre-/*mdx* (*n* = 5–6), and C57 wild-type (*n* = 1) quadriceps were incubated with 1:500 MMP14 (Abcam, ab51074), 1:100 periostin (Abcam, ab152099), or 1:200 Lrrc39 (Bioss Antibodies, bs-12312R) primary antibodies followed by 1:200 Alexa Fluor 555 goat anti-rabbit IgG (Thermofisher, A21429). Images were taken on a Nikon Eclipse 800 or NiE microscope equipped with a Moment CMOS camera using Nikon Elements software. For the tenascin C, MMP14, periostin, and Lrrc39 staining, images were quantified using Adobe Photoshop. Briefly, areas expressing the protein of interest were highlighted in blue while areas that were not part of the tissue were colored white. The percentage area containing the protein of interest was calculated by dividing the area highlighted blue by the total non-white area. One-way ANOVAs with Tukey’s or Dunnett’s multiple comparisons test, as appropriate for each data set were used to compare the differences in the percentage area of muscle tissue containing tenascin C between 8- week-old and 1-year-old Cre-/*mdx* and LysM-MRcko/*mdx* quadriceps, and between 8-week-old Cre-/*mdx*, LysM-MRcko/*mdx*, and MCK-MRcko/*mdx* quadriceps. One-year-old MCK-MRcko/*mdx* mice and samples were not available, but we have previously published staining of these samples with fibronectin (Hauck et al., 2019). One-way ANOVAs were also used to compare the differences in the percentage area of muscle tissue containing MMP14, periostin, and Lrrc39 between quadriceps from 8-week-old Cre-/*mdx*, LysM-MRcko/*mdx*, and MCK-MRcko/*mdx* mice. A significant difference was defined by a *p*-value of less than 0.05.

### Western blots

Western blots were performed to test protein levels corresponding to select gene expression changes identified from the microarray. Protein was isolated from flash-frozen quadriceps from 8-week-old LysM-MRcko/*mdx*, MCK-MRcko/*mdx*, Cre-/*mdx*, C57 wild-type, and *mdx* mice and resuspended in Newcastle buffer (4M Urea, 3.8% SDS, 74 mM Tris pH 6.8, 20% glycerol) as previously described (Chadwick et al., 2015). 100 μg of total protein from each sample was run on 10% (for Lox and for C57/*mdx* validation of all proteins) or 8% (for periostin and MMP14) SDS-PAGE and transferred to nitrocellulose. Total protein was marked using the Ponceau S reagent (Sigma, P7170-1L) before antibody probing and was used as an additional normalization control. Membranes were incubated in rabbit primary antibodies against periostin (1:1000, Novus Bio, NBP1-30042), MMP14 (1:700, Abcam, ab51074), and Lox (1:500, Invitrogen, MA5-32817) or glyceraldehyde-3-phosphate dehydrogenase (GAPDH, 1:10,000, Proteintech, 10494-1-AP) as a housekeeping control. Goat anti-rabbit horseradish peroxidase (HRP) (Jackson ImmunoResearch Labs, 111-035-144) was used as secondary antibody. HRP was detected using an ECL2 kit (Pierce, 80196) followed by exposure to film and the Chemidoc MP Imaging system (BioRad). Quantification was performed using the ImageLab software (BioRad). We used GAPDH as a housekeeping protein control for Postn and MMP14, but since Lox (32 kDa) and GAPDH (36 kDa) were of similar size, we used total protein as a normalization control for Lox. Briefly, band intensities were calculated by the software and normalized to the housekeeping GAPDH control bands and/or to total protein via Ponceau S staining for proteins too similar in size to GAPDH.

## Results

### Myeloid MR knockout increases proinflammatory and profibrotic gene expression

**FIGURE 1:**
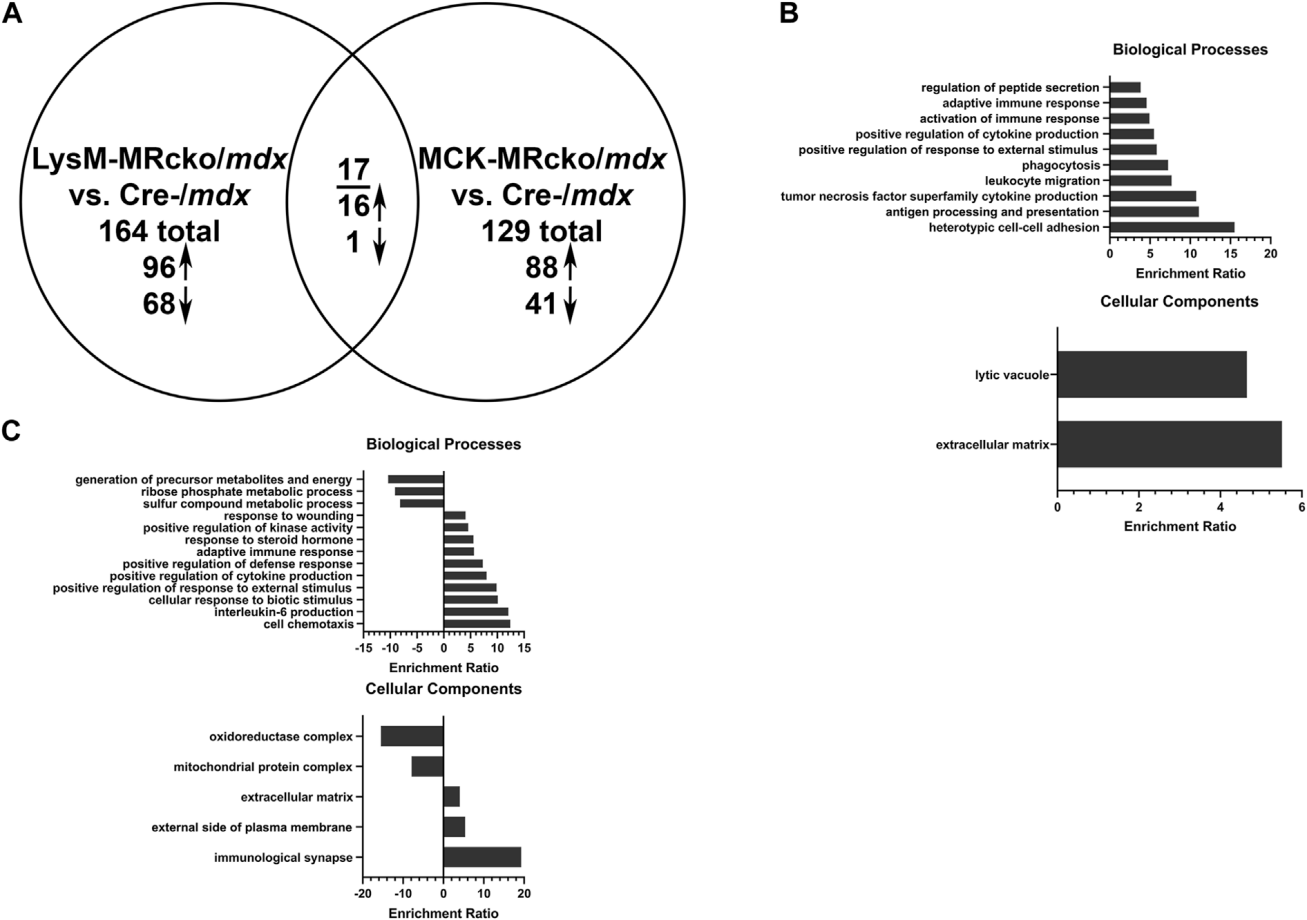
Myeloid cell MRcko/*mdx* increased proinflammatory and profibrotic skeletal muscle gene expression compared to myofiber MRcko/*mdx*. **(A)** Differentially expressed genes from gene expression microarray comparing quadriceps muscles from 8-week-old myeloid cell (LysM) MRcko/*mdx*, myofiber (MCK) MRcko/*mdx*, and Cre-/*mdx* mice. Ninety-six genes were increased, and 68 genes were decreased in LysM-MRcko/*mdx* compared to Cre-/*mdx*. Eighty-eight genes were increased, and 41 genes were decreased in MCK-MRcko/*mdx* compared to Cre-/*mdx*. Seventeen genes were conserved between LysM-MRcko/*mdx* and MCK-MRcko/*mdx*, with 16 increased and one decreased in both knockouts compared to Cre-/*mdx*. **(B)** Gene ontology overrepresentation (GO) analysis for biological processes and cellular components for the LysM-MRcko/*mdx* vs. Cre-/*mdx* comparison. **(C)** GO analysis for biological processes and cellular components for the MCK-MRcko/*mdx* vs. Cre-/*mdx* comparison.

Gene expression microarray analysis was conducted on quadriceps muscles from 8-week-old dystrophin-deficient mice with a myeloid-specific knockout of MR (LysM-MRcko/*mdx*) or a myofiber-specific knockout of MR (MCK-MRcko/*mdx*), compared to *mdx* control littermates. Expression of 164 genes was changed in the LysM-MRcko/*mdx* muscles compared to *mdx* controls, with 96 genes increased and 68 decreased (Figure 1A; Supplementary Table S1). Expression of 129 genes was changed in the MCK-MRcko/*mdx* muscles compared to *mdx* controls, with 88 increased and 41 decreased (Figure 1A; Supplementary Table S2). Seventeen genes were conserved between the two knockouts compared to *mdx* controls, with 16 of those increased with MR knockout. Only one gene, *Adgrf5*, was reduced in both knockouts (Figure 1A; Table 1). Gene ontology overrepresentation analysis showed that biological processes involved in the immune response and inflammation and the cellular component of extracellular matrix were most highly enriched in the myeloid cell MR knockout (LysM-MRcko/*mdx*) compared to Cre-/*mdx* mice (Figure 1B). More varied biological and cellular processes were altered in the myofiber MR knockout (MCK-MRcko/*mdx*) compared to Cre-/*mdx* (Figure 1C).

**TABLE 1:** Myofibroblast cluster genes upregulated in myeloid MRcko/*mdx* quadriceps compared to Cre-/*mdx* and myofiber MRcko/*mdx* quadriceps muscles.

Functional categories	Gene	Full gene name	Fold change (LysM-MRcko/*mdx* vs. Cre-/*mdx*)	Fold change (LysM-MRcko/*mdx* vs. MCK-MRcko/*mdx*)
Apoptosis	*Dap*	*death-associated protein*	2.2	2.1
Cytoskeleton	*Tubb2b*	*tubulin, beta 2B class IIB*	2.8	2.3
	*Pfn1*	*profilin 1*	**X**	2.5
Electron Transport Chain	*Cox6a1*	*cytochrome c oxidase subunit VIa polypeptide 1*	2.1	2.9
Extracellular Space	*Loxl1*	*lysyl oxidase-like 1*	3.0	2.1
	*Mmp14*	*matrix metalloproteinase 14*	4.1	2.3
	*Mfap4*	*microfibrillar-associated protein 4*	**X**	2.3
	*Fn1*	*fibronectin 1*	**X**	2.3
	*Postn*	*periostin*	3.4	2.4
	*Hspg2*	*heparan sulfate proteoglycan 2*	**X**	3.1
	*Cilp*	*cartilage intermediate layer protein*	2.6	3.5
	*Tnc*	*tenascin C*	5.5	6.3
Inflammatory Response	*C1qtnf3*	*C1q and tumor necrosis factor related protein 3*	4.9	4.0
Nitric Oxide Generation	*Ddah1*	*dimethylarginine dimethylaminohydrolase 1*	**X**	2.3
Signal	*Sulf2*	*sulfatase 2*	**X**	2.1
	*Ncam1*	*neural cell adhesion molecule 1*	**X**	2.6
	*Olfml2b*	*olfactomedin-like 2B*	2.9	3.7

Genes were functionally categorized using the Database for Annotation, Visualization, and Integrated Discovery (DAVID). Positive fold changes indicate that gene expression was increased in LysM-MRcko/*mdx* compared to either Cre-/*mdx* controls or MCK-MRcko/*mdx* and *vice versa*. Bold X’s signify a lack of 2-fold gene expression change in the LysM-MRcko/*mdx* vs. Cre-/*mdx* comparison.

**FIGURE 2:**
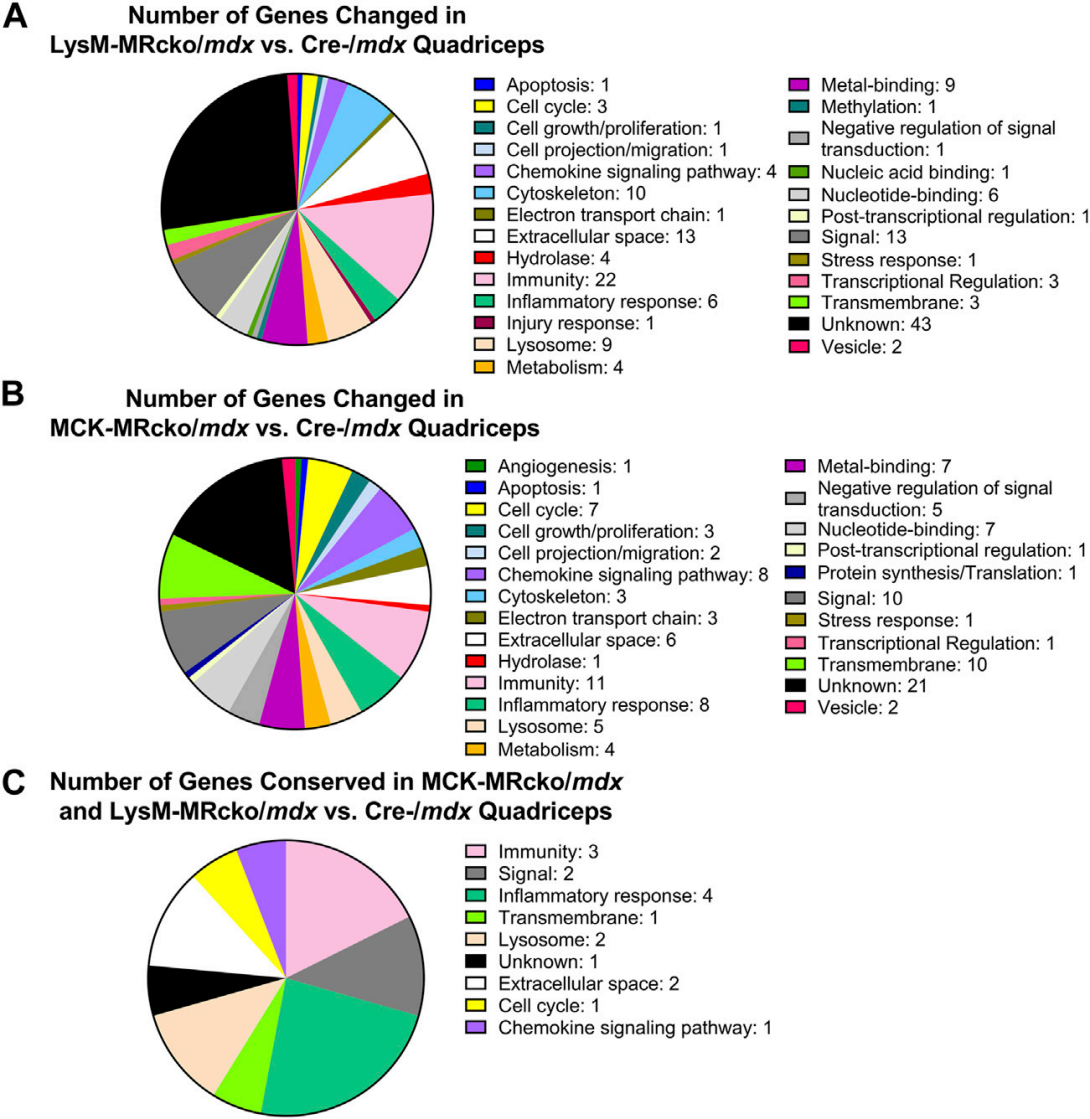
DAVID analysis of differentially expressed genes from the gene expression microarray. **(A)** Distribution of DAVID gene categories for the LysM-MRcko/*mdx* vs. Cre-/*mdx* comparison. **(B)** Distribution of DAVID gene categories for the MCK-MRcko/*mdx* vs. Cre-/*mdx* comparison. **(C)** Distribution of DAVID gene categories for the 17 genes conserved between the LysM-MRcko/*mdx* vs. Cre-/*mdx* and MCK-MRcko/*mdx* vs. Cre-/*mdx* comparisons.

Functional categories from the gene expression microarray were determined using DAVID analysis. Genes affecting fibrosis were predominantly found in the extracellular space category including *Tnc*, *Postn*, and *Lox*, but several genes found in other categories also have known roles in fibrosis, such as *Spp1*, *Lilrb4a*, and *Anxa1* (Supplementary Tables S1, S2). The myeloid cell MR knockout led to differential expression of 28 genes with roles in the immunity and inflammatory response categories, and 13 genes in the extracellular space category (Figure 2A). The myofiber MR knockout included 6 genes that were changed in the extracellular space category and 19 genes changed in the immunity and inflammatory response categories (Figure 2B). Due to the predominance of increased extracellular matrix binding genes in the molecular functions GO category of the myeloid MRcko/*mdx* muscle, we compared the upregulated genes in these mice to published datasets of fibroblast genes (Buechler et al., 2021). We found that 10 myofibroblast signature genes were upregulated in LysM-MRcko/*mdx* compared to Cre-/*mdx*, including *Postn*, *Mmp14*, and *Loxl1*, and 17 myofibroblast genes were increased in LysM-MRcko/*mdx* compared to MCK-MRcko/*mdx*, additionally including *Fn1* and *Hspg2* (Table 1). We also examined the functional categories of the gene expression changes that are conserved between the myofiber and myeloid MR knockouts. Seven of the 17 conserved genes were involved in inflammation and immunity, while two of the genes were in the extracellular space category (Figure 2C; Table 2).

**TABLE 2:** Conserved differentially expressed genes between LysM-MRcko/*mdx* versus Cre-/*mdx* and MCK-MRcko/*mdx* versus Cre-/*mdx* comparisons.

Functional categories	Conserved genes	Full gene name	Fold change (LysM-MRcko/*mdx* vs. Cre-/*mdx*)	Fold change (MCK-MRcko/*mdx* vs. Cre-/*mdx*)
Cell cycle	*S100a6*	*S100 calcium binding protein A6 (calcyclin)*	2.0	2.2
Chemokine signaling pathway	*Pf4*	*platelet factor 4*	2.1	3.3
Extracellular Space	*Mmp12*	*matrix metallopeptidase 12*	3.3	3.0
	*Lox*	*lysyl oxidase*	2.2	2.5
Immunity	*Adgre1*	*adhesion G protein-coupled receptor E1*	2.5	2.2
	*C3ar1*	*complement component 3a receptor 1*	3.4	2.5
	*Itgam*	*integrin alpha M*	2.2	2.2
	*Cd84*	*CD84 antigen*	2.1	3.5
Inflammatory Response	*Spp1*	*secreted phosphoprotein 1*	2.9	10.0
	*Il1rn*	*interleukin 1 receptor antagonist*	2.0	2.2
	*Tlr13*	*toll-like receptor 13*	2.0	2.7
Lysosome	*Lgmn*	*legumain*	2.5	2.3
	*Ctss*	*cathepsin S*	2.3	2.5
Signal	*Folr2*	*folate receptor 2*	2.2	2.6
	*Adgrf5*	*adhesion G protein-coupled receptor F5*	−2.4	−3.2
Unknown	*Gm14699*	*predicted gene 14699*	2.5	2.5
Transmembrane	*Cd53*	*CD53 antigen*	2.1	3.2

Genes were functionally categorized using the Database for Annotation, Visualization, and Integrated Discovery (DAVID). Positive fold changes indicate that gene expression was increased in the MR knockouts compared to Cre-/*mdx* controls and *vice versa*.

### Tenascin C deposition decreases with age in myeloid MR knockout and cre-/*mdx*

**FIGURE 3:**
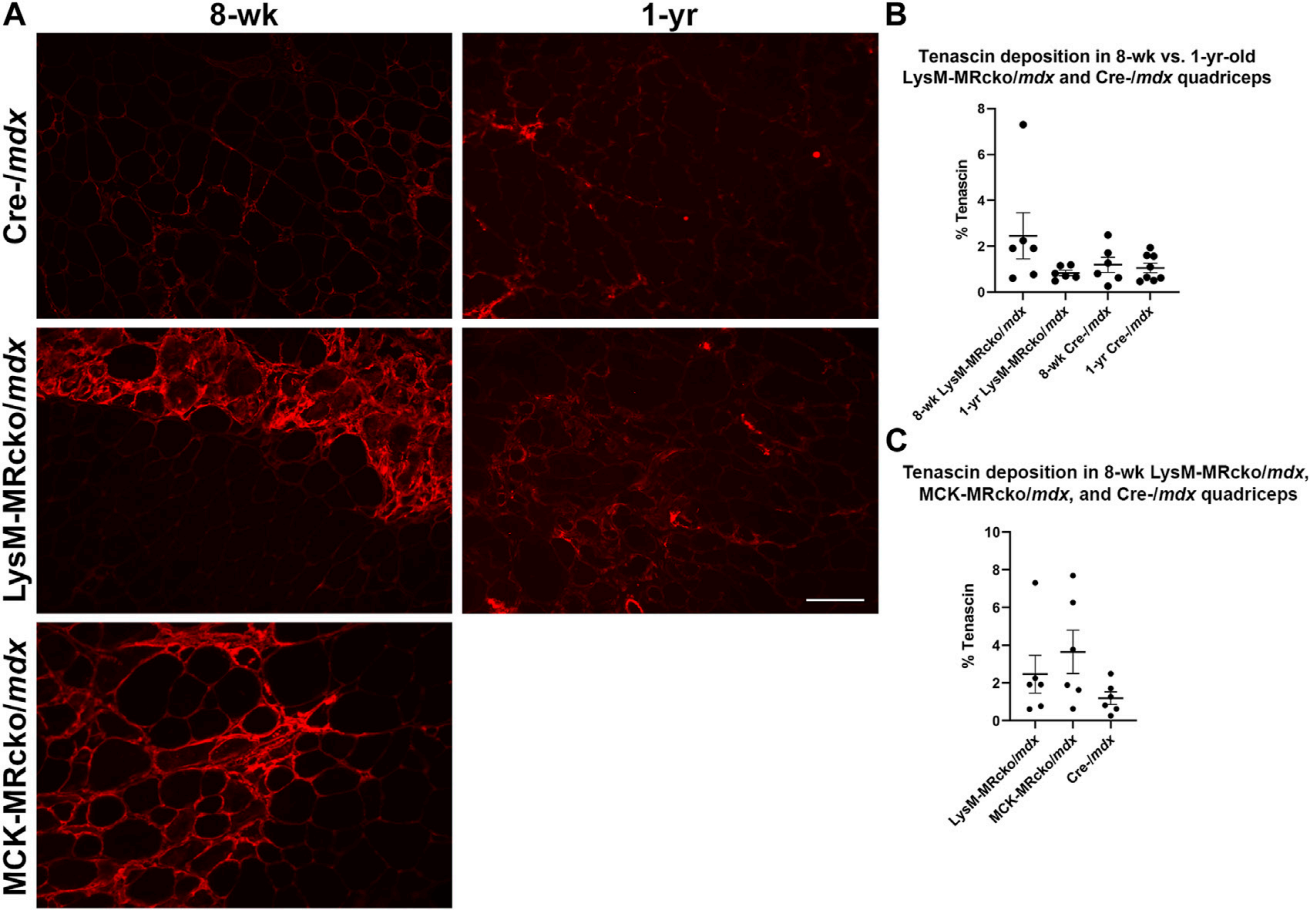
Tenascin C localization in young and old dystrophic quadriceps muscles. **(A)** Immunofluorescence staining of tenascin C in quadriceps muscles from 8-week-old LysM-MRcko/*mdx*, Cre-/*mdx*, MCK-MRcko/*mdx* and 1-year-old LysM-MRcko/*mdx* and Cre-/*mdx* mice. Bar = 100 µm. **(B)** Quantification of the percentage of quadriceps muscle area containing tenascin C staining in 8-week-old and 1-year-old LysM-MRcko/*mdx* and Cre-/*mdx* mice. **(C)** Quantification of the percentage of quadriceps muscle area containing staining in 8-week-old LysM-MRcko/*mdx*, Cre-/*mdx*, and MCK-MRcko/*mdx*. One-way ANOVAs with either Tukey’s **(B)** or Dunnett’s **(C)** multiple comparisons test revealed no significant differences between groups.

Tenascin C (Tnc) is an extracellular matrix component known to be present in fibrotic scars. Tnc plays a role in regulating fibrosis causing its persistence in multiple organs, including skin, lung, heart, liver, and cornea (El-Karef et al., 2007; Carey et al., 2010; Sumioka et al., 2013; Shimojo et al., 2015; Bhattacharyya et al., 2016). In skeletal muscle, Tnc is localized mostly to areas of damage and accompanies inflammation and fibrosis during wound healing (Flück et al., 2003; Flück et al., 2008). *Tnc* expression was increased in myeloid cell MR knockout 5.5-fold compared to Cre-/*mdx* quadriceps and 6.3-fold compared to myofiber MR knockout but was not different between myofiber MR knockout and Cre-/*mdx* quadriceps. Immunofluorescence staining shows an upward trend of the percentage area of muscle tissue containing tenascin C protein deposition in 8-week-old LysM-MRcko/*mdx* compared to Cre-/*mdx* quadriceps muscles (Figure 3). We investigated whether tenascin C localization changes as fibrosis accumulates with age and aged LysM-MRcko/*mdx* mice to 1 year. Tenascin C staining appears less prevalent in quadriceps muscles at 1 year compared to 8 weeks-of-age, and one-way ANOVA with Tukey’s multiple comparisons test shows that there is no significant difference in tenascin C deposition in quadriceps from all four groups (Figures 3A, B). Additionally, we compared tenascin C localization between 8-week-old LysM-MRcko/*mdx*, MCK-MRcko/*mdx*, and Cre-/*mdx* quadriceps. One-way ANOVA with Dunnett’s multiple comparisons test showed no significant differences between genotypes in the areas stained for tenascin C (Figures 3A, C).

### Loxl1, but not Lox, co-localizes with collagen in fibrotic regions of dystrophic skeletal muscle

**FIGURE 4:**
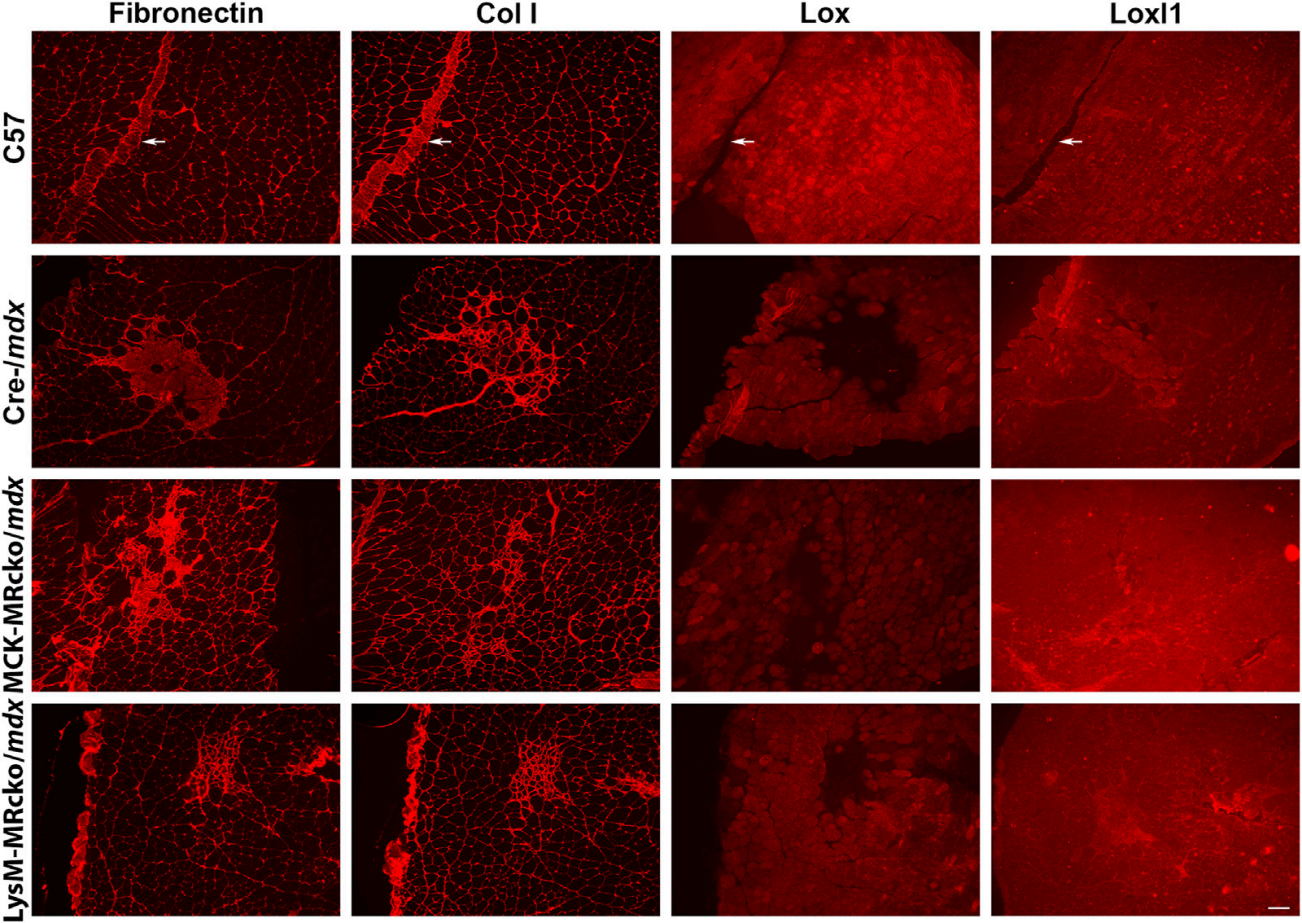
Lox localizes within myofibers and not to fibrotic areas in dystrophic muscles. Immunofluorescence staining of fibronectin, type I collagen (Col I), Lox, and Loxl1 on serial sections of quadriceps muscles from 8-week-old C57 wild-type, Cre-/*mdx*, MCK-MRcko/*mdx*, and LysM-MRcko/*mdx* mice. Fibronectin, Col I, and Loxl1 co-localize to fibrotic regions present in dystrophic muscle, but Lox localizes only in the cytoplasm of myofibers. Fibronectin and Col I, but not Loxl1, also localize to blood vessels (white arrows) and surround each myofiber providing support to the tissue in C57. Bar = 100 µm.

**FIGURE 5:**
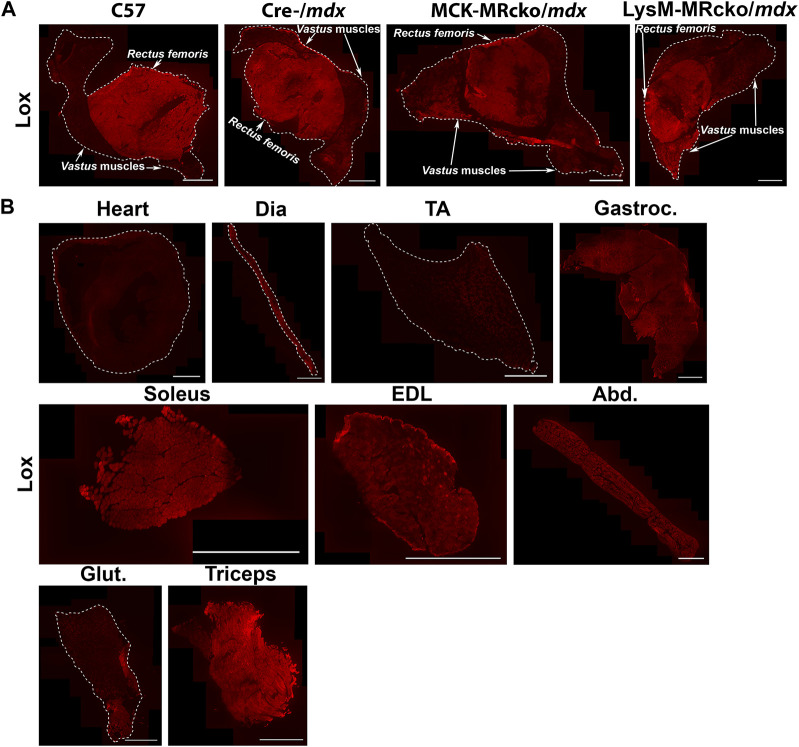
Lox shows a unique pattern of staining in quadriceps and other muscles. **(A)** Immunofluorescence staining of Lox in quadriceps from 8-week-old C57 wild-type, Cre-/*mdx*, MCK-MRcko/*mdx* and LysM-MRcko/*mdx* mice. Lox staining is concentrated in the *rectus femoris* circular head of wild-type quadriceps muscles and shows some additional lower-level staining in the *vastus* heads from the three dystrophic mice. Bar = 1 mm. The *rectus femoris* and *vastus* heads of the quadriceps are labeled with white arrows and text. **(B)** Immunofluorescence staining of Lox in heart, diaphragm, *tibialis anterior*, gastrocnemius, soleus, *extensor digitorum longus*, abdominals, *gluteus maximus*, and triceps from 8-week-old C57 wild-type mice. Lox shows different localization patterns in different skeletal muscles. Bar = 1 mm. White dashed lines show the borders of the muscle sections for those with no detectable staining near the borders.

*Lox*, encoding lysyl oxidase, is one of the genes increased in both the myeloid cell and myofiber MR knockouts compared to *mdx* control skeletal muscle (Table 2). Lox is one of a family of five enzymes, including lysyl oxidase like 1-4 (Loxl1-4), that have been shown to crosslink type I collagens to help stabilize and strengthen the extracellular matrix (Clarke et al., 2013). Since *Lox* was increased in both knockouts, even though myofiber and myeloid knockouts have opposite effects on dystrophic muscle fibrosis (Hauck et al., 2019; Howard et al., 2022b), we next investigated Lox protein localization. We performed immunofluorescence staining of quadriceps muscles from 8-week-old myofiber and myeloid MRcko/*mdx* mice (Figures 4, 5A). In both knockouts, *mdx* and C57 wild-type control mice, Lox localizes cytoplasmically within myofibers and does not colocalize with collagen or fibronectin in areas of fibrosis in dystrophic muscles (Figure 4). Furthermore, Lox localizes to all myofibers from the *rectus femoris* of the quadriceps, but not to myofibers in other heads of the quadriceps muscle from wild-type mice (Figure 5A). More myofiber staining in these other quadriceps regions is present in *mdx* quadriceps muscles and is even more prevalent in myofiber and myeloid MRcko/*mdx* quadriceps (Figure 5A). Staining of longitudinal sections of *mdx* and C57 wild-type quadriceps revealed that Lox localization is present at consistent levels within fibers of the *rectus femoris* and is not increased near the myotendinous junction in either the *rectus femoris* or *vastus* muscles (Supplementary Figure S1). We then stained for Lox in other wild-type C57 skeletal muscles and observed uniform Lox myofiber localization in the diaphragm, gastrocnemius, soleus, *extensor digitorum longus*, abdominals, and triceps (Figure 5B). Lox localization was restricted to small discrete regions of the *tibialis anterior* and *gluteus maximus* (Figure 5B). In the heart, Lox was not present cytoplasmically in cardiomyocytes (Figure 5B) and localized only to intercalated discs (Supplementary Figure S1).

Gene expression of *Loxl1* was increased in myeloid MRcko/*mdx* compared to both *mdx* and myofiber MRcko/*mdx* mice. Therefore, we investigated Loxl1 localization in quadriceps muscles. Loxl1 colocalizes with fibronectin and type I collagen in fibrotic areas, but not with Lox (Figure 4), supporting that the Loxl1 family member may be the one involved in collagen crosslinking in dystrophic skeletal muscle fibrosis.

### Protein levels do not correlate with gene expression differences for representative fibrotic proteins

**FIGURE 6:**
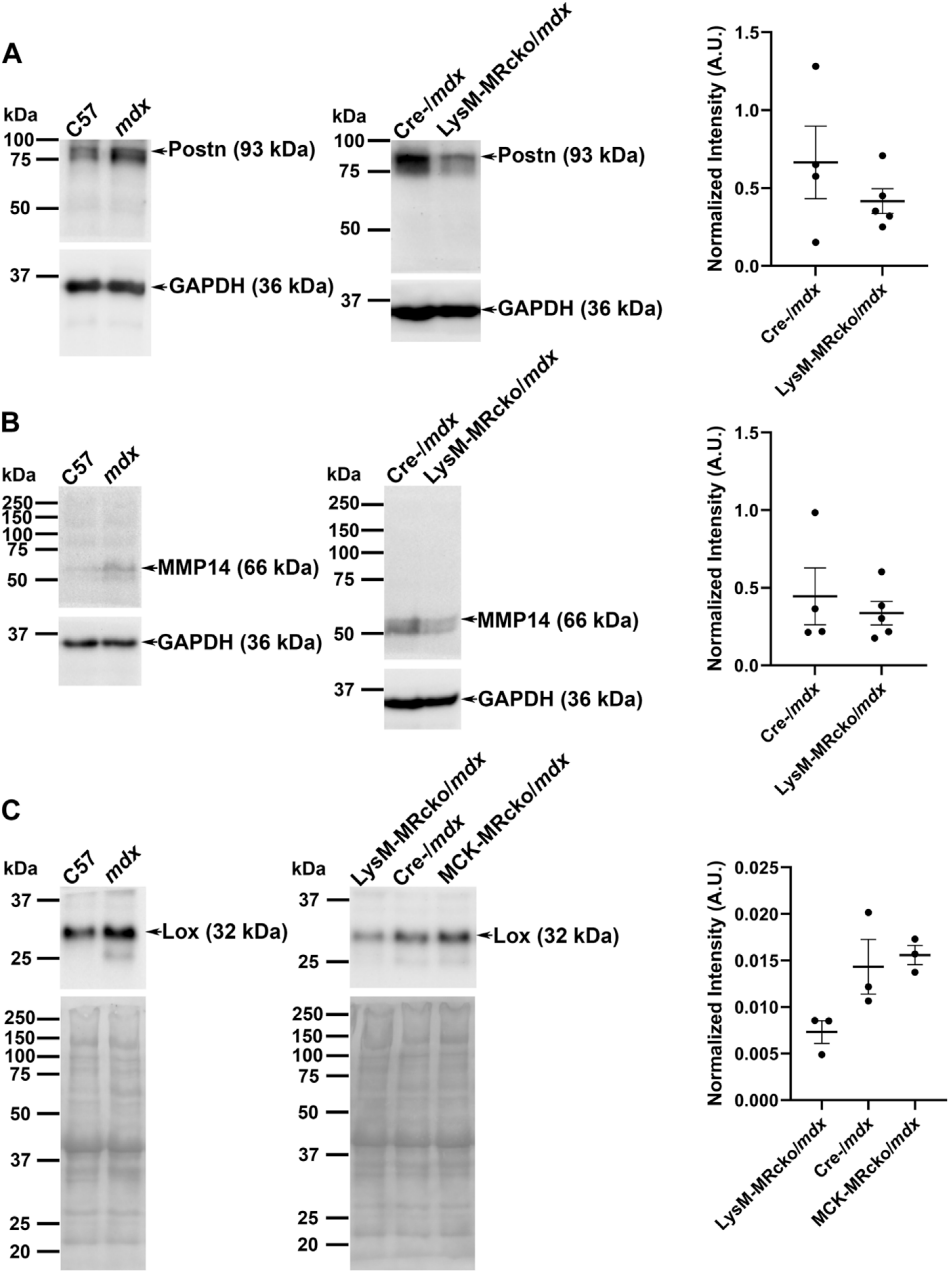
Western blot analyses of periostin, MMP14, and Lox. **(A)** Periostin (93 kDa), **(B)** MMP14 (66 kDa), and **(C)** Lox (32 kDa) antibodies were first validated on quadriceps protein homogenates from 8-week-old C57 wild-type and *mdx* mice (left panels) and then tested on quadriceps protein samples from 8 week-old LysM-MRcko/*mdx* and Cre-/*mdx* mice **(A,B)** or LysM-MRcko/*mdx*, Cre-/*mdx* mice and MCK-MRcko/*mdx* samples **(C)** (center panels). GAPDH (36 kDa) was used as a normalization control for periostin and MMP 14. Total protein via Ponceau S staining was used for normalization for Lox protein levels. Quantification of band intensities for each sample after normalization is shown [**(A–C)** right panels] for each Western blot (arbitrary units). Unpaired t-tests with Welch’s correction (Postn, MMP14) and one-way ANOVA with Dunnett’s multiple comparisons test (Lox) showed no significant differences in protein expression for any of the proteins.

Matrix metalloproteinase 14 (MMP14) is a known modulator of collagen deposition and the organization of the extracellular matrix (Zigrino et al., 2016). Periostin is secreted by activated myofibroblasts (Kanisicak et al., 2016). In our gene expression microarray, *MMP14* was increased by 4-fold and *periostin* (*Postn*) was increased by 3-fold in LysM-MRcko/*mdx* compared to Cre-/*mdx* (Table 1). To test whether gene expression changes for *MMP-14* and *Postn* in addition to *Lox* corresponded to protein level changes, we assessed their protein products by western analysis. We first validated the antibodies for MMP-14 and periostin by comparing them between C57 wild-type and more fibrotic dystrophic *mdx* quadriceps muscle homogenates. Postn and MMP14 both showed increased protein levels in *mdx*, though expression was similar between the two genotypes for Lox, as expected due to the wide-spread localization of Lox in quadriceps myofibers (Figures 6A–C). Given that *Postn* and *MMP14* gene expression were significantly different between LysM-MRcko/*mdx* and Cre-/*mdx*, we then performed western analysis on samples from these two genotypes (Figures 6A, B). Since *Lox* gene expression was significantly increased in both LysM-MRcko/*mdx* and MCK-MRcko/*mdx* quadriceps (Supplementary Tables S1, S2), we tested Lox protein levels in all three genotypes (Figure 6C). Unpaired t-tests with Welch’s correction for periostin and MMP14 showed no significant protein level differences between genotypes. One-way ANOVA with Dunnett’s multiple comparisons test showed no significant differences between genotypes for Lox protein levels.

### MMP14, periostin, and Lrrc39 localize to fibrotic areas in skeletal muscles

**FIGURE 7:**
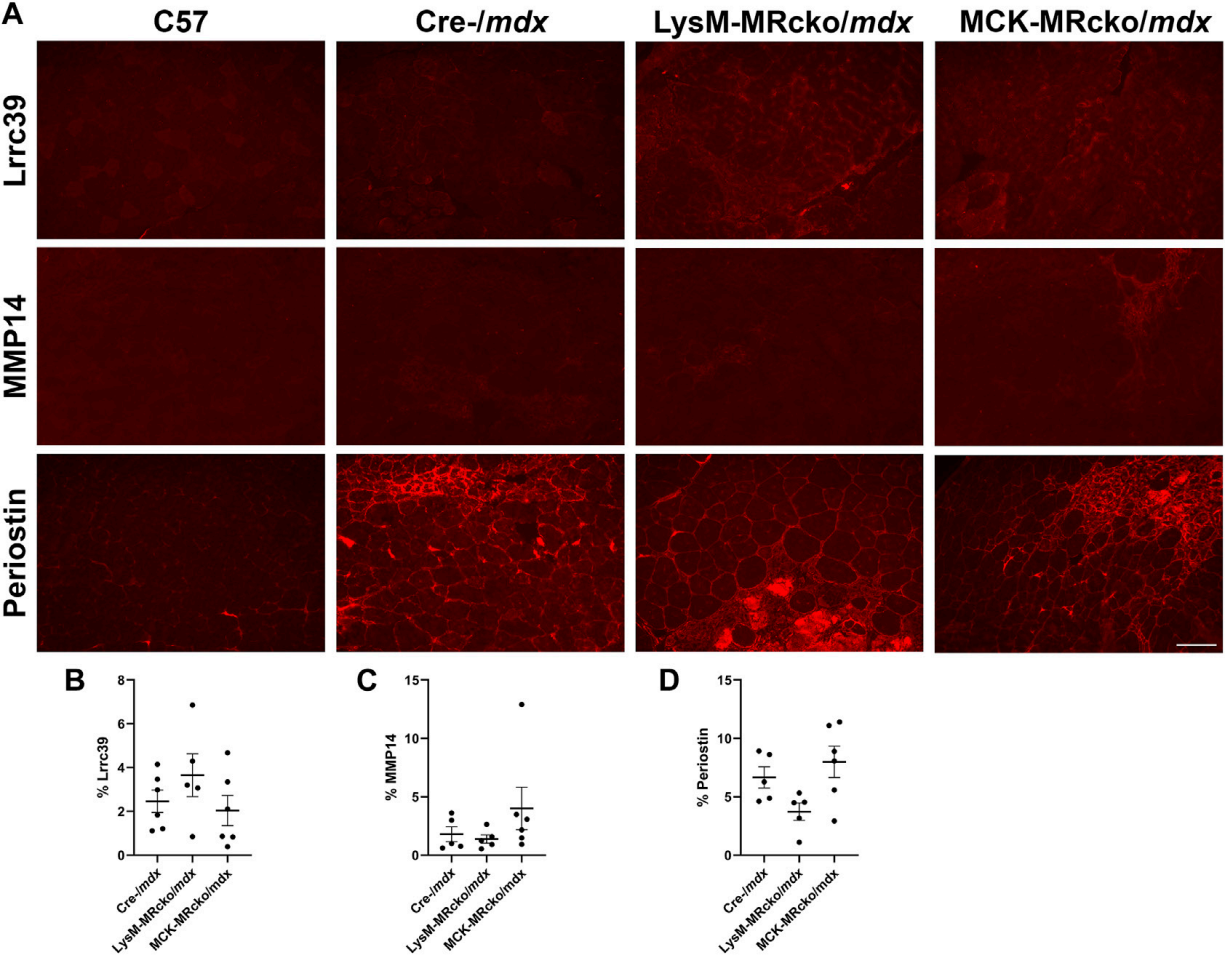
Lrrc39, MMP14, and periostin are present in fibrotic areas of dystrophic skeletal muscles. **(A)** Immunofluorescence staining of Lrrc39, MMP14, and periostin in quadriceps muscles from 8-week-old C57 wild-type, Cre-/*mdx*, LysM-MRcko/*mdx*, and MCK-MRcko/*mdx* mice. Bar = 100 µm. **(B–D)** Quantification of the percentage area stained with Lrrc39, MMP14, and periostin, respectively. One-way ANOVA with Dunnett’s multiple comparisons test showed that there were no significant differences in expression for any of the three proteins.

We then assessed the localization of MMP-14 and periostin to confirm their presence in fibrotic regions of muscles. We also investigated localization of Lrrc39. *Lrrc39* gene expression was decreased by 2-fold in LysM-MRcko/*mdx* compared to Cre-/*mdx* (Supplementary Table S1) and had also previously been identified to be increased in dystrophic muscles after 2 weeks of lisinopril and spironolactone (LS) treatment (Chadwick et al., 2017). Lrrc39 plays a role in myofibril organization and stretch sensing (Will et al., 2010). We performed immunofluorescence staining of quadriceps from 8-week-old C57 wild-type, Cre-/*mdx*, LysM-MRcko/*mdx*, and MCK-MRcko/*mdx* mice using antibodies against Lrrc39, periostin and MMP14. All three proteins were observed to localize to fibrotic areas present in Cre-/*mdx*, LysM-MRcko/*mdx*, and MCK-MRcko/*mdx* quadriceps muscles, but were absent from C57 wild-type quadriceps (Figure 7A). One-way ANOVA with Dunnett’s multiple comparisons test showed no significant differences in the percent of section area containing localization of MMP-14, periostin or Lrrc39 between the Cre-/*mdx*, LysM-MRcko/*mdx*, and MCK-MRcko/*mdx* genotypes (Figures 7B–D).

## Discussion

Increased expression of profibrotic and proinflammatory genes in the myeloid cell MR knockout coupled with higher levels of fibrosis compared to Cre-/*mdx* skeletal muscle suggests MR-regulated crosstalk between the different cell types in the dystrophic muscle microenvironment. The contrasting reduction in inflammation and fibrosis gene expression in myofiber MR knockout *mdx* compared to Cre-/*mdx* mice also supports this crosstalk.

Eight-week-old LysM-MRcko/*mdx* quadriceps showed increases of 17 myofibroblast signature genes, including *Postn*, *Fn1*, *Tnc*, and *Loxl1* (Buechler et al., 2021). Periostin, fibronectin, and tenascin C are components of fibrotic scars, while lysyl oxidase-like 1 is a member of the lysyl oxidase family and has a role in collagen crosslinking (Clarke et al., 2013; Smith et al., 2016). Conversely, myofiber MR knockout led to upregulation of genes involved in antifibrotic and anti-inflammatory responses, such as *leukocyte immunoglobulin-like receptor, subfamily B, members 4A* and *4B*, *Lilrb4a* and *Lilrb4b*, both orthologs of human *Lilrb4*. *Lilrb4* is expressed by immune cells in various tissues and negatively regulates the immune response. A recent study has shown that suppression of *Lilrb4* led to increased inflammation, fibrosis, and apoptosis in the heart (Li et al., 2019). *Anxa1* is another gene with anti-inflammatory activity upregulated in myofiber MR knockouts. In skeletal muscle, Annexin A1 has important roles in promoting regeneration by stimulating satellite cell migration and differentiation, and in converting macrophages from the pro-inflammatory M1 phenotype to the reparative M2 phenotype (Bizzarro et al., 2010; McArthur et al., 2020). Another example, Hepatitis A virus *cellular receptor 2* (*Havcr2*), is expressed by T helper 1 (Th1) cells and by a population of T regulatory (Treg) cells that are known to infiltrate skeletal muscle after acute injury (Burzyn et al., 2013). The Treg cells appear to play a role in converting M1 macrophages to M2 macrophages, which leads to reduced muscle damage (Burzyn et al., 2013). *Havcr2* is also known to inhibit Th1 activity and regulate macrophage activity to promote immune tolerance (Chen et al., 2023).

Both knockouts show that there are indirect effects of MR signaling from non-fibroblast cell types on the function and behavior of fibroblasts. Fibroblasts represent the main cell type in the body that produces and manipulates extracellular matrix, so the changes in profibrotic gene expression seen in the two MR knockouts support that disrupting MR signaling in myeloid cells or myofibers leads to contrasting changes in fibroblasts.

Comparison with previous microarray data suggests that these MR knockouts differentially affect gene expression compared to MR antagonism. We have performed gene expression microarray on quadriceps from dystrophin deficient, utrophin haplo-insufficient (*mdx*;*utrn* ^*+/−*^) mice that were treated for 2 weeks with angiotensin-converting enzyme (ACE) inhibitor lisinopril plus either the MRA spironolactone or eplerenone (Chadwick et al., 2017). We have also performed gene expression microarray on quadriceps from *mdx*;*utrn* ^*+/−*^ mice treated for 16 weeks with lisinopril and spironolactone (Chadwick et al., 2015). Given that the myeloid cell and myofiber MR knockouts only inhibit MR in specific cell types, we compared gene expression changes from the two knockouts to the changes that occurred due to MR antagonism throughout the muscle microenvironment. The similarities and differences in gene expression changes between these data could help elucidate genes targeted by MR and help explain the mechanism by which MR regulates gene expression. Two MR regulated genes that may be contributing to the fibrotic phenotype in the LysM mice are *Lrtm1* (*Leucine rich repeats and transmembrane domains 1*) and *Lrrc39* (*Leucine rich repeat containing 39*), which were decreased in LysM-MRcko/*mdx* (3- and 2.1-fold, respectively) and increased after 2 weeks of lisinopril and spironolactone (LS) treatment (2.5- and 2.1-fold, respectively) (Chadwick et al., 2017). *Cyp2e1* was increased in LysM-MRcko/*mdx* (3.7-fold) and increased by 16 weeks of LS treatment (4.0-fold) and therefore represents a potential MR target regulated in myeloid cells (Chadwick et al., 2015). Both *Lrtm1* and *Lrrc39* have roles in skeletal muscle function. A recent study has shown that *Lrtm1* is highly expressed during myoblast differentiation and skeletal muscle regeneration after injury, playing an important role in regulating myoblast differentiation (Li et al., 2020). *Lrrc39* encodes a member (Lrrc39/myomasp) of the sarcomeric M-band, which serves as an anchor for the thick filaments of the myofibril. It has been shown that the M-band is a major hub for signaling within the muscle fiber in addition to its role as a structural anchor, and Lrrc39 was found to help regulate stretch sensing (Will et al., 2010). Biomechanical stress was found to significantly downregulate *Lrrc39* expression, and *in vitro* and *in vivo* knockdown of *Lrrc39* led to significant force reduction and contractile dysfunction (Will et al., 2010). The observation that expression of *Lrtm1* and *Lrrc39* are decreased in the myeloid cell MR knockout, but increased after MRA treatment is consistent with benefits in muscle force production and membrane stability with MRA treatment, and increased fibrosis in myeloid MRcko/*mdx* mice (Chadwick et al., 2016; Chadwick et al., 2017; Hauck et al., 2019; Howard et al., 2022a; Howard et al., 2022b). *Lrrc39* was also decreased in *mdx*;*utrn* ^*+/−*^ mice after 2 weeks of treatment with the glucocorticoid prednisolone, which also leads to increased muscle fibrosis in *mdx* mice (Chadwick et al., 2017).

*Tenascin C* (*Tnc*) was one of the most highly upregulated genes in the myeloid cell MR knockout. Tnc is known to be present in fibrotic scars (Flück et al., 2008) and seems to be more plentiful at younger ages. Its deposition trends upwards in 8-week-old LysM-MRcko/*mdx* quadriceps compared to both 8-week-old Cre-/*mdx* quadriceps and 1-year-old LysM-MRcko/*mdx* mice. It is also known to play a role in newt limb regrowth after injury, so it may be more prevalent in skeletal muscle at a younger age when the muscle is more regenerative (Onda et al., 1991; Calve et al., 2010). If the deposition of Tnc at a younger age could be replicated at a later stage of the disease, it is possible that the higher levels of Tnc could lead to a matrix that is more permissible for ongoing muscle regeneration or even for reversing fibrosis.

*Lysyl oxidase* (*Lox*) is one of the genes upregulated with both myeloid and myofiber MR knockout compared to *mdx* controls. Lox is usually defined as a collagen crosslinker and therefore localized to the extracellular matrix (Clarke et al., 2013; Smith et al., 2016). A recent spatial transcriptomics study also identified markers of fibrosis and regeneration in the more severe D2-*mdx* mouse model, and this data demonstrated that *Lox* is expressed highly in fibrotic regions of dystrophic muscle compared to non-fibrotic regions (Heezen et al., 2023). *Lox* has been implicated as a potential therapeutic target for treatment of fibrosis in skin, lung, and skeletal muscle (Clarke et al., 2013; Smith et al., 2016; Huang et al., 2019). However, Lox protein is not observed in fibrotic regions and only appears within myofibers in both wild-type and dystrophic muscles. This data explains why a previous study testing a Lox inhibitor as a treatment for fibrosis in *mdx* mice did not result in any benefit (Brashear et al., 2022). In addition, Lox, to our knowledge, is the only protein that localizes specifically to myofibers only in the *rectus femoris* head of wild-type quadriceps and not to the *vastus* muscles. We have also shown that Lox localization throughout myofibers longitudinally appears uniform and is not enriched near myotendinous junctions.

Previous studies have shown Lox localization within myofibers during muscle development and regeneration (Kutchuk et al., 2015; Gabay Yehezkely et al., 2020). Lox has also been shown to act as a promoter of myoblast differentiation via oxidation of *vestigial like 3* (*Vgll3*), a co-activator required for activating *myogenic enhancer factor 2* (*Mef2*) to stimulate myogenesis (Gabay Yehezkely et al., 2020). Global *Lox* knockouts also show defects in fetal muscle development, with reduced myofiber numbers and disorganization (Kutchuk et al., 2015). Since the MCK-MRcko/*mdx* mice recapitulate the improved muscle force production of MRA treatment, it is possible that Lox may play a role in this improved contractile force (Hauck et al., 2019). Contractile force has not yet been measured for the LysM-MRcko/*mdx* mice, so Lox upregulation may confer this benefit, despite increased fibrosis (Howard et al., 2022b). However, the exact function of Lox in skeletal muscle and an explanation for its unique pattern between muscle types that is not fiber-type-specific, will need to be investigated in the future. Loxl1, in contrast with Lox, does colocalize with fibronectin and type I collagen in fibrotic areas. Loxl1 is also an important regulator of collagen crosslinking and has been implicated in several inflammatory and fibrotic diseases (Ohmura et al., 2012; Smith et al., 2016; Ma et al., 2018; Schlötzer-Schrehardt and Zenkel, 2019), so it is likely acting as a collagen crosslinker in dystrophic skeletal muscle. To determine whether our observations of localization of Lox and Loxl1 agree with publicly available RNA sequencing datasets, we investigated single-cell spatial transcriptomics performed in D2-*mdx* mice (Stec et al., 2023). This dataset showed that *Lox* and *Loxl1* were both expressed in fibroblasts. However, our observations are based on protein localization, which may not correlate with RNA expression.

Immunofluorescence staining of periostin and MMP14, two of the most significantly changed genes between LysM-MRcko/*mdx* and Cre-/*mdx* quadriceps, showed that both proteins localize to fibrotic areas, but protein levels were not significantly different between genotypes. Immunofluorescence staining of Lrrc39 in 8-week-old quadriceps from C57, LysM-MRcko/*mdx*, and MCK-MRcko/*mdx* mice showed that Lrrc39 is also present in fibrotic areas of skeletal muscles, but is also not significantly different. We attempted to test protein levels of other genes that demonstrated expression differences, including Loxl1, Lrrc39, and tenascin C, but the antibodies tested did not yield specific bands of the expected sizes. It is well known that protein levels do not always correlate with gene expression levels, but some possible explanations for this discrepancy include a feedback loop between gene expression and protein synthesis, the small number of samples that can be loaded in a gel for Western blotting, or discrepancies with the ability to detect and quantify protein levels. It is possible that the gene targets we have investigated may be involved in a negative feedback loop with their respective proteins, wherein high protein levels can inhibit gene expression and conversely gene expression can be enhanced when the protein expression is low due to turnover or production (Singh, 2011). One possibility is that downstream signaling from MR in myeloid cells alters fibroblast apoptosis and leads to increased fibrotic gene expression in surviving fibroblasts, but this hypothesis will require future investigation.

A potential limitation of this study is that the myeloid cell MR conditional knockout is not restricted to striated muscle and affects all myeloid lineage cells in the body. It is possible that changes in MR signaling from myeloid cells outside the muscle could affect the muscle microenvironment indirectly due to potential impacts on other organ systems. Further investigation of protein levels, localization, and function from additional gene expression changes identified in this study that are common or different between MCK-MRcko/*mdx* and LysM-MRcko/*mdx* may identify MR-regulated signals between myofiber, myeloid, and fibroblast cell types that regulate the dystrophic muscle microenvironment. MR-regulated genes leading to secreted proteins may also provide new biomarkers to monitor and optimize the clinical use of MRA treatment for DMD.

## Scope statement

We show for the first time that conditional knockout of mineralocorticoid receptors (MR) in myofibers and myeloid cells have contrasting effects on gene expression in the dystrophic skeletal muscle microenvironment. Inhibition of MR signaling with MR antagonists has previously shown clinical efficacy in Duchenne muscular dystrophy (DMD) cardiomyopathy and preclinical efficacy in skeletal muscle in DMD models. Myeloid MR knockout led to increases in myofibroblast gene expression, supporting MR-regulated crosstalk between inflammatory cells and fibroblasts. Surprisingly, *lysyl oxidase* (*Lox*), canonically a collagen crosslinker, was increased in both myofiber and myeloid MR knockouts, but did not localize to fibrotic regions of dystrophic skeletal muscle. Lox localized within myofibers, including only a region of quadriceps muscles. *Lysyl oxidase like 1* (*Loxl1*), another Lox family member, was increased only in myeloid MR knockout muscle and Loxl1 localized specifically to fibrotic regions, supporting Loxl1 as a new anti-fibrotic target in muscle. Both myofiber and myeloid MR knockout changed expression of genes involved in inflammation. Fibrosis and inflammation exacerbate pathology of muscular dystrophies, so defining signals between cell-types in the striated muscle microenvironment will ultimately be required for optimal therapeutic approaches for muscle diseases.

## Data Availability

The data presented in the study are deposited in the NCBI Gene Expression Omnibus repository, accession number GSE244569 (https://www.ncbi.nlm.nih.gov/geo/query/acc.cgi?acc=GSE244569) and summarized in the Supplementary Material.
