# Supplementary material for: Cell-type specific effects of mineralocorticoid receptor gene expression suggest intercellular communication regulating fibrosis in skeletal muscle disease
Source: Front Physiol. 2024 Apr 26;15:1322729. doi: 10.3389/fphys.2024.1322729 (PMC11082420; doi:10.3389/fphys.2024.1322729)
Supplement: Supplementary file 1 [file DataSheet2.PDF]

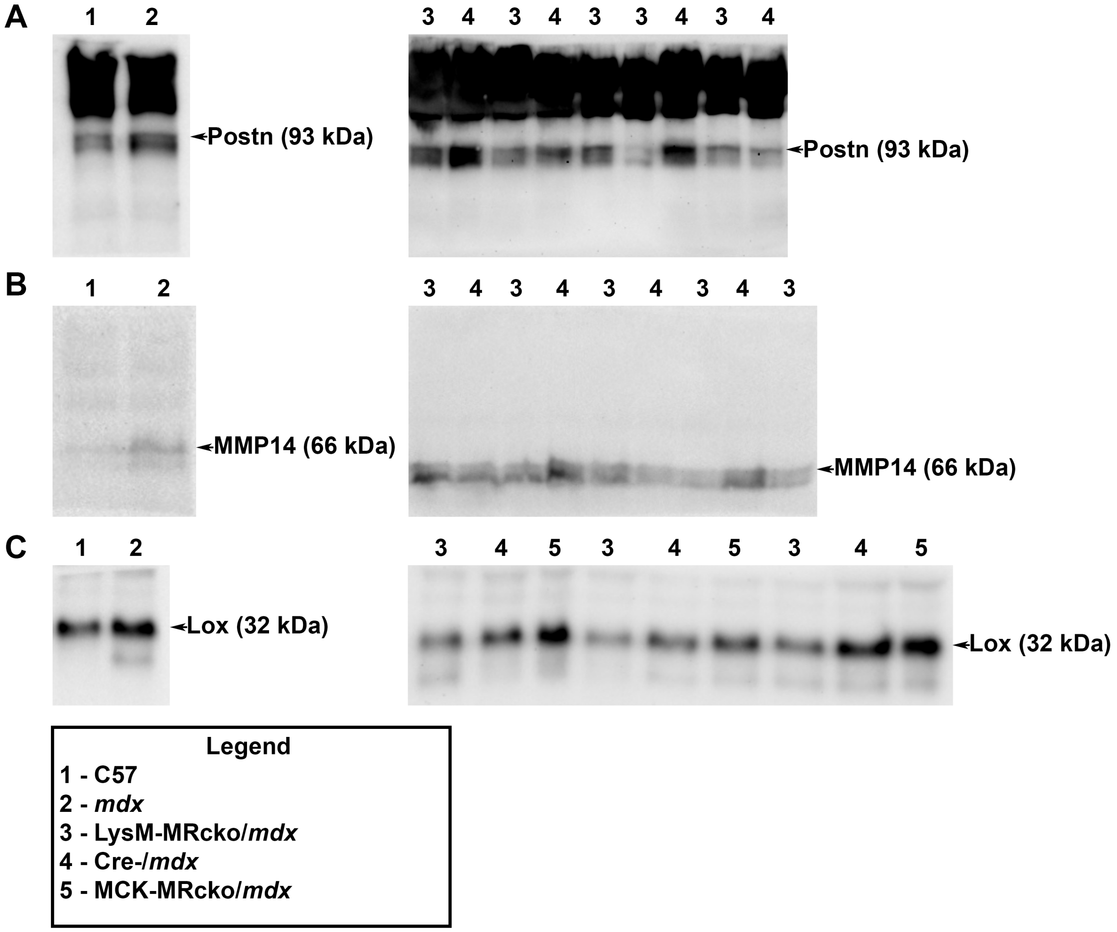

Supplemental Figure 2. Whole western blots containing all samples used to validate and test periostin, MMP14, and Lox. (**A**) Western blot for periostin (93 kDa) performed using 8-week-old C57 and *mdx* samples (**left**) and 8-week-old LysM-MRcko/*mdx* and Cre-/*mdx* samples (**right**). (**B**) Western blot for MMP14 (66 kDa) performed using 8-week-old C57 and *mdx* samples (**left**) and 8-week-old LysM-MRcko/*mdx* and Cre-/*mdx* samples (**right**). (**C**) Western blot for Lox (32 kDa) performed using 8-week-old C57 and *mdx* samples (**left**) and 8-week-old LysM-MRcko/*mdx*, Cre-/*mdx*, and MCK-MRcko/*mdx* samples (**right**).
