# Supplementary material for: Cell-type specific effects of mineralocorticoid receptor gene expression suggest intercellular communication regulating fibrosis in skeletal muscle disease
Source: Front Physiol. 2024 Apr 26;15:1322729. doi: 10.3389/fphys.2024.1322729 (PMC11082420; doi:10.3389/fphys.2024.1322729)
Supplement: Supplementary file 2 [file DataSheet3.docx]

**Supplemental Table 1.** Differentially expressed genes between 8-week-old LysM-MRcko/*mdx* and Cre-/*mdx* quadriceps muscles. Genes were functionally categorized using the Database for Annotation, Visualization, and Integrated Discovery (DAVID). Positive fold changes indicate that gene expression was increased in LysM-MRcko/*mdx* compared to Cre-/*mdx* controls and vice versa.

| **Functional Categories** | **Gene** | **Full Gene Name** | **Fold Change (LysM-MRcko/*mdx* vs. Cre-/*mdx*)** | **P-val** | **FDR P-val** |
| --- | --- | --- | --- | --- | --- |
| Apoptosis | *Dap* | *death-associated protein* | 2.2 | 0.014 | 0.785 |
| Cell cycle | *S100a6* | *S100 calcium binding protein A6 (calcyclin)* | 2.0 | 0.020 | 0.785 |
|  | *Ppp2r3a* | *protein phosphatase 2, regulatory subunit B, alpha* | -2.2 | 0.007 | 0.785 |
|  | *Map2k6* | *mitogen-activated protein kinase kinase 6* | -2.2 | 0.002 | 0.780 |
| Cell growth/proliferation | *Emp3* | *epithelial membrane protein 3* | 2.5 | 0.009 | 0.785 |
| Cell projection/migration | *Coro6* | *coronin 6* | -3.3 | 0.017 | 0.785 |
| Chemokine signaling pathway | *Cxcl16* | *chemokine (C-X-C motif) ligand 16* | 2.2 | 0.005 | 0.785 |
|  | *Pf4* | *platelet factor 4* | 2.1 | 0.025 | 0.785 |
|  | *Cx3cr1* | *chemokine (C-X3-C motif) receptor 1* | 2.1 | 0.047 | 0.785 |
|  | *Amot* | *angiomotin* | -2.2 | 0.000 | 0.675 |
| Cytoskeleton | *Tubb6* | *tubulin, beta 6 class V* | 2.9 | 0.017 | 0.785 |
|  | *Tubb2b* | *tubulin, beta 2B class IIB* | 2.8 | 0.012 | 0.785 |
|  | *Grn* | *granulin* | 2.7 | 0.027 | 0.785 |
|  | *Tuba1a* | *tubulin, alpha 1A* | 2.7 | 0.007 | 0.785 |
|  | *Tuba1c* | *tubulin, alpha 1C* | 2.2 | 0.008 | 0.785 |
|  | *Tubb2a* | *tubulin, beta 2A class IIA* | 2.2 | 0.045 | 0.785 |
|  | *Nckap1l* | *NCK associated protein 1 like* | 2.1 | 0.003 | 0.780 |
|  | *Tuba1b* | *tubulin, alpha 1B* | 2.1 | 0.011 | 0.785 |
|  | *Gm21857; LOC100861837; Mid1* | *predicted gene, 21857; midline 1* | 2.0 | 0.047 | 0.785 |
|  | *Dmd* | *dystrophin, muscular dystrophy* | -2.5 | 0.035 | 0.785 |
| Electron transport chain | *Cox6a1* | *cytochrome c oxidase subunit VIa polypeptide 1* | 2.1 | 0.003 | 0.785 |
| Extracellular space | *Tnc* | *tenascin C* | 5.5 | 0.001 | 0.711 |
|  | *Lgals3bp* | *lectin, galactoside-binding, soluble, 3 binding protein* | 4.2 | 0.010 | 0.785 |
|  | *Mmp14* | *matrix metallopeptidase 14 (membrane-inserted)* | 4.1 | 0.014 | 0.785 |
|  | *Postn* | *periostin, osteoblast specific factor* | 3.4 | 0.001 | 0.780 |
|  | *Mmp12* | *matrix metallopeptidase 12* | 3.3 | 0.034 | 0.785 |
|  | *Loxl1* | *lysyl oxidase-like 1* | 3.0 | 0.002 | 0.780 |
|  | *Cilp* | *cartilage intermediate layer protein, nucleotide pyrophosphohydrolase* | 2.6 | 0.001 | 0.711 |
|  | *Tgfbi* | *transforming growth factor, beta induced* | 2.5 | 0.001 | 0.711 |
|  | *Lrrc15* | *leucine rich repeat containing 15* | 2.2 | 0.000 | 0.675 |
|  | *Lox* | *lysyl oxidase* | 2.2 | 0.007 | 0.785 |
|  | *Anpep* | *alanyl (membrane) aminopeptidase* | 2.2 | 0.003 | 0.785 |
|  | *Mgp* | *matrix Gla protein* | 2.1 | 0.032 | 0.785 |
|  | *Fsd2* | *fibronectin type III and SPRY domain containing 2* | -2.1 | 0.004 | 0.785 |
| Hydrolase | *Pld3* | *phospholipase D family, member 3* | 2.3 | 0.003 | 0.785 |
|  | *Man2b2* | *mannosidase 2, alpha B2* | 2.2 | 0.001 | 0.711 |
|  | *Nudt12* | *nudix (nucleoside diphosphate linked moiety X)-type motif 12* | -2.1 | 0.015 | 0.785 |
|  | *Acot2* | *acyl-CoA thioesterase 2* | -4.4 | 0.012 | 0.785 |
| Immunity | *C3ar1* | *complement component 3a receptor 1* | 3.4 | 0.002 | 0.780 |
|  | *C1qb* | *complement component 1, q subcomponent, beta polypeptide* | 3.4 | 0.015 | 0.785 |
|  | *H2-Ab1* | *histocompatibility 2, class II antigen A, beta 1* | 2.9 | 0.018 | 0.785 |
|  | *Itgb2* | *integrin beta 2* | 2.9 | 0.006 | 0.785 |
|  | *Csf1r* | *colony stimulating factor 1 receptor* | 2.6 | 0.000 | 0.675 |
|  | *C4b* | *complement component 4B (Chido blood group)* | 2.5 | 0.006 | 0.785 |
|  | *Mpeg1* | *macrophage expressed gene 1* | 2.5 | 0.003 | 0.785 |
|  | *Ly86* | *lymphocyte antigen 86* | 2.5 | 0.023 | 0.785 |
|  | *Adgre1* | *adhesion G protein-coupled receptor E1* | 2.5 | 0.003 | 0.785 |
|  | *Cfb* | *complement factor B* | 2.4 | 0.002 | 0.780 |
|  | *Unc93b1* | *unc-93 homolog B1 (C. elegans)* | 2.4 | 0.003 | 0.783 |
|  | *C1qc* | *complement component 1, q subcomponent, C chain* | 2.4 | 0.005 | 0.785 |
|  | *Itgax* | *integrin alpha X* | 2.4 | 0.008 | 0.785 |
|  | *Fcer1g* | *Fc receptor, IgE, high affinity I, gamma polypeptide* | 2.3 | 0.013 | 0.785 |
|  | *Itgam* | *integrin alpha M* | 2.2 | 0.000 | 0.675 |
|  | *H2-K1* | *histocompatibility 2, K1, K region* | 2.2 | 0.010 | 0.785 |
|  | *Gm11710; Gm11711; Cd300lh* | *predicted gene 11710; CD300 antigen like family member H* | 2.2 | 0.021 | 0.785 |
|  | *Cd84* | *CD84 antigen* | 2.1 | 0.032 | 0.785 |
|  | *Tlr9* | *toll-like receptor 9* | 2.0 | 0.003 | 0.783 |
|  | *Ccdc134* | *coiled-coil domain containing 134* | 2.0 | 0.020 | 0.785 |
|  | *Mr1* | *major histocompatibility complex, class I-related* | -2.1 | 0.017 | 0.785 |
|  | *Cd59a* | *CD59a antigen* | -2.9 | 0.022 | 0.785 |
| Inflammatory response | *C1qtnf3* | *C1q and tumor necrosis factor related protein 3* | 4.9 | 0.000 | 0.675 |
|  | *Spp1* | *secreted phosphoprotein 1* | 2.9 | 0.008 | 0.785 |
|  | *Pld4* | *phospholipase D family, member 4* | 2.8 | 0.001 | 0.780 |
|  | *P2ry6* | *pyrimidinergic receptor P2Y, G-protein coupled, 6* | 2.1 | 0.003 | 0.785 |
|  | *Tlr13* | *toll-like receptor 13* | 2.0 | 0.007 | 0.785 |
|  | *Il1rn* | *interleukin 1 receptor antagonist* | 2.0 | 0.029 | 0.785 |
| Injury response | *Tmem8c* | *transmembrane protein 8C* | 6.1 | 0.013 | 0.785 |
| Lysosome | *Cd68* | *CD68 antigen* | 3.7 | 0.002 | 0.780 |
|  | *Ifi30* | *interferon gamma inducible protein 30* | 3.5 | 0.005 | 0.785 |
|  | *Hexa* | *hexosaminidase A* | 2.9 | 0.003 | 0.783 |
|  | *Laptm5* | *lysosomal-associated protein transmembrane 5* | 2.6 | 0.017 | 0.785 |
|  | *Ctsz* | *cathepsin Z* | 2.5 | 0.003 | 0.780 |
|  | *Lgmn* | *legumain* | 2.5 | 0.003 | 0.785 |
|  | *Ctss* | *cathepsin S* | 2.3 | 0.003 | 0.780 |
|  | *Hexb* | *hexosaminidase B* | 2.1 | 0.011 | 0.785 |
|  | *Acp5* | *acid phosphatase 5, tartrate resistant* | 2.0 | 0.012 | 0.785 |
| Metabolism | *Cyp2e1* | *cytochrome P450, family 2, subfamily e, polypeptide 1* | 3.7 | 0.010 | 0.785 |
|  | *Cbr2* | *carbonyl reductase 2* | 2.2 | 0.016 | 0.785 |
|  | *Pgam1-ps2* | *phosphoglycerate mutase 1, pseudogene 2* | 2.1 | 0.022 | 0.785 |
|  | *Ppargc1a* | *peroxisome proliferative activated receptor, gamma, coactivator 1 alpha* | -2.5 | 0.033 | 0.785 |
| Metal-binding | *Tnnt2* | *troponin T2, cardiac* | 3.0 | 0.017 | 0.785 |
|  | *Mir692-3; Ftl1; Ftl2-ps* | *microRNA 692-3; ferritin light chain 1; ferritin light polypeptide 2, pseudogene* | 2.2 | 0.033 | 0.785 |
|  | *Tnni1* | *troponin I, skeletal, slow 1* | 2.1 | 0.007 | 0.785 |
|  | *Anxa5* | *annexin A5* | 2.0 | 0.012 | 0.785 |
|  | *Esyt1* | *extended synaptotagmin-like protein 1* | 2.0 | 0.007 | 0.785 |
|  | *Abcb7* | *ATP-binding cassette, sub-family B (MDR/TAP), member 7* | -2.0 | 0.006 | 0.785 |
|  | *Zfp931* | *zinc finger protein 931* | -2.1 | 0.003 | 0.785 |
|  | *Zim1* | *zinc finger, imprinted 1* | -2.2 | 0.016 | 0.785 |
|  | *Slc25a12* | *solute carrier family 25 (mitochondrial carrier, Aralar), member 12* | -3.3 | 0.005 | 0.785 |
| Methylation | *Coq5* | *coenzyme Q5 homolog, methyltransferase (yeast)* | -2.9 | 0.001 | 0.780 |
| Negative regulation of signal transduction | *Sirpa* | *signal-regulatory protein alpha* | 2.0 | 0.009 | 0.785 |
| Nucleic acid binding | *Tigd4* | *tigger transposable element derived 4* | -2.1 | 0.026 | 0.785 |
| Nucleotide-binding | *Hist2h3c2; Hist2h3b* | *histone cluster 2, H3c2; histone cluster 2, H3b* | 2.2 | 0.042 | 0.785 |
|  | *Ptbp1* | *polypyrimidine tract binding protein 1* | 2.1 | 0.008 | 0.785 |
|  | *Gatc* | *glutamyl-tRNA(Gln) amidotransferase, subunit C* | -2.2 | 0.007 | 0.785 |
|  | *Hint3* | *histidine triad nucleotide binding protein 3* | -2.2 | 0.006 | 0.785 |
|  | *Papss2* | *3-phosphoadenosine 5-phosphosulfate synthase 2* | -2.5 | 0.027 | 0.785 |
|  | *Uprt* | *uracil phosphoribosyltransferase (FUR1) homolog (S. cerevisiae)* | -2.9 | 0.004 | 0.785 |
| Post-transcriptional regulation | *Mirlet7f-1* | *microRNA let7f-1* | -2.4 | 0.003 | 0.785 |
| Signal | *Gpnmb* | *glycoprotein (transmembrane) nmb* | 5.0 | 0.047 | 0.785 |
|  | *Fcrls* | *Fc receptor-like S, scavenger receptor* | 4.3 | 0.013 | 0.785 |
|  | *Olfml2b* | *olfactomedin-like 2B* | 2.9 | 0.020 | 0.785 |
|  | *Siglec1* | *sialic acid binding Ig-like lectin 1, sialoadhesin* | 2.5 | 0.003 | 0.785 |
|  | *Mup20* | *major urinary protein 20* | 2.4 | 0.050 | 0.785 |
|  | *Mup-ps7* | *major urinary protein, pseudogene 7* | 2.2 | 0.019 | 0.785 |
|  | *Folr2* | *folate receptor 2 (fetal)* | 2.2 | 0.031 | 0.785 |
|  | *Usp15* | *ubiquitin specific peptidase 15* | -2.0 | 0.004 | 0.785 |
|  | *Grasp* | *GRP1 (general receptor for phosphoinositides 1)-associated scaffold protein* | -2.1 | 0.018 | 0.785 |
|  | *Prex2* | *phosphatidylinositol-3,4,5-trisphosphate-dependent Rac exchange factor 2* | -2.3 | 0.010 | 0.785 |
|  | *Pkia* | *protein kinase inhibitor, alpha* | -2.3 | 0.006 | 0.785 |
|  | *Adgrf5* | *adhesion G protein-coupled receptor F5* | -2.4 | 0.044 | 0.785 |
|  | *Lrtm1* | *leucine-rich repeats and transmembrane domains 1* | -3.0 | 0.002 | 0.780 |
| Stress response | *Lrrc39* | *leucine rich repeat containing 39* | -2.1 | 0.002 | 0.780 |
| Transcriptional Regulation | *Spi1* | *spleen focus forming virus (SFFV) proviral integration oncogene* | 2.6 | 0.006 | 0.785 |
|  | *Nr1d2* | *nuclear receptor subfamily 1, group D, member 2* | -2.0 | 0.012 | 0.785 |
|  | *Abra* | *actin-binding Rho activating protein* | -3.7 | 0.038 | 0.785 |
| Transmembrane | *Ms4a7* | *membrane-spanning 4-domains, subfamily A, member 7* | 2.2 | 0.025 | 0.785 |
|  | *Cd53* | *CD53 antigen* | 2.1 | 0.046 | 0.785 |
|  | *Atad1* | *ATPase family, AAA domain containing 1* | -2.7 | 0.006 | 0.785 |
| Unknown | *Gm6682* | *predicted gene 6682; tubulin, alpha 1C pseudogene* | 2.5 | 0.015 | 0.785 |
|  | *Gm14699* | *predicted gene 14699* | 2.5 | 0.003 | 0.783 |
|  | *Gm11709* | *predicted gene 11709* | 2.4 | 0.009 | 0.785 |
|  |  |  | 2.3 | 0.026 | 0.785 |
|  | *Gm5424* | *predicted gene 5424* | 2.2 | 0.005 | 0.785 |
|  | *Gm8910* | *predicted gene 8910* | 2.1 | 0.028 | 0.785 |
|  | *Gm11710; Gm11711; Cd300lh* | *predicted gene 11710; predicted gene 11711; CD300 antigen like family member H* | 2.1 | 0.020 | 0.785 |
|  | *Ms4a14* | *PREDICTED: membrane-spanning 4-domains, subfamily A, member 14* | 2.1 | 0.045 | 0.785 |
|  | *Gm14326* | *predicted gene 14326* | -2.0 | 0.010 | 0.785 |
|  | *Gm4724; 0610010B08Rik* | *predicted gene 4724; RIKEN cDNA 0610010B08 gene* | -2.0 | 0.024 | 0.785 |
|  | *Gm14420* | *predicted gene 14420* | -2.0 | 0.005 | 0.785 |
|  | *Gm14421* | *predicted gene 14421* | -2.0 | 0.013 | 0.785 |
|  | *Gm14418* | *predicted gene 14418* | -2.1 | 0.033 | 0.785 |
|  | *Gm14431; Gm8898; Gm4723* | *predicted gene 14431;*  *predicted gene 8898;*  *predicted gene 4723;* | -2.1 | 0.014 | 0.785 |
|  | *Gm20274* | *predicted gene, 20274* | -2.1 | 0.013 | 0.785 |
|  | *Gm14431; Gm8898* | *predicted gene 14431; predicted gene 8898* | -2.1 | 0.019 | 0.785 |
|  | *Gm14431; Gm8898; Gm4245* | *predicted gene 14431; predicted gene 8898; predicted gene 4245* | -2.1 | 0.019 | 0.785 |
|  | *Gm14434; 0610010B08Rik* | *predicted gene 14434; RIKEN cDNA 0610010B08 gene* | -2.1 | 0.017 | 0.785 |
|  | *Gm14297* | *predicted gene 14297* | -2.1 | 0.034 | 0.785 |
|  | *Gm14307* | *predicted gene 14307* | -2.1 | 0.034 | 0.785 |
|  | *Gm16359* | *predicted gene 16359 [Source:MGI Symbol;Acc:MGI:3840119]* | -2.1 | 0.034 | 0.785 |
|  | *Gm14308* | *predicted gene 14308* | -2.1 | 0.023 | 0.785 |
|  | *Gm2026* | *predicted gene 2026* | -2.1 | 0.012 | 0.785 |
|  | *AW549877* | *expressed sequence AW549877* | -2.2 | 0.032 | 0.785 |
|  | *Gm14409* | *predicted gene 14409* | -2.2 | 0.007 | 0.785 |
|  | *Gm14430* | *predicted gene 14430* | -2.2 | 0.023 | 0.785 |
|  | *Gm1818* | *predicted gene 1818* | -2.2 | 0.030 | 0.785 |
|  | *Gm14322* | *predicted gene 14322* | -2.2 | 0.014 | 0.785 |
|  | *Etohi1* | *ethanol induced 1* | -2.2 | 0.026 | 0.785 |
|  | *Gm14305* | *predicted gene 14305* | -2.2 | 0.030 | 0.785 |
|  | *Gm14306* | *predicted gene 14306* | -2.2 | 0.021 | 0.785 |
|  | *Gm14431; Gm8898* | *predicted gene 14431; predicted gene 8898* | -2.3 | 0.010 | 0.785 |
|  | *Gm2004* | *predicted gene 2004* | -2.3 | 0.027 | 0.785 |
|  | *F830016B08Rik* | *RIKEN cDNA F830016B08 gene* | -2.3 | 0.006 | 0.785 |
|  | *Gm14327* | *predicted gene 14327* | -2.3 | 0.035 | 0.785 |
|  | *Gm14288; Gm14440* | *predicted gene 14288; predicted gene 14440* | -2.4 | 0.018 | 0.785 |
|  | *Gm14419* | *predicted gene 14419* | -2.4 | 0.025 | 0.785 |
|  | *Gm14288; Gm14435* | *predicted gene 14288; predicted gene 14435* | -2.4 | 0.026 | 0.785 |
|  | *Snord64* | *small nucleolar RNA, C/D box 64* | -2.4 | 0.029 | 0.785 |
|  | *Gm14288; Gm14440* | *predicted gene 14288; predicted gene 14440* | -2.5 | 0.022 | 0.785 |
|  | *Lsmem1* | *leucine-rich single-pass membrane protein 1* | -2.5 | 0.008 | 0.785 |
|  | *Gm14295* | *predicted gene 14295* | -2.5 | 0.033 | 0.785 |
|  | *Gm4841* | *predicted gene 4841* | -2.6 | 0.009 | 0.785 |
| Vesicle | *Vat1* | *vesicle amine transport protein 1 homolog (T californica)* | 2.1 | 0.009 | 0.785 |
|  | *Snx12* | *sorting nexin 12* | -2.2 | 0.026 | 0.785 |

**Supplemental Table 2.** Differentially expressed genes between 8-week-old MCK-MRcko/*mdx* and Cre-/*mdx* quadriceps muscles. Genes were functionally categorized using the Database for Annotation, Visualization, and Integrated Discovery (DAVID). Positive fold changes indicate that gene expression was increased in MCK-MRcko/*mdx* compared to Cre-/*mdx* controls and vice versa.

| **Functional Categories** | **Gene** | **Full Gene Name** | **Fold Change (MCK-MRcko/*mdx* vs. Cre-/*mdx*)** | **P-val** | **FDR P-val** |
| --- | --- | --- | --- | --- | --- |
| Angiogenesis | *Rras* | *Harvey rat sarcoma oncogene, subgroup R* | -3.1 | 0.044 | 0.843 |
| Apoptosis | *Ppif* | *peptidylprolyl isomerase F (cyclophilin F)* | -2.0 | 0.010 | 0.843 |
| Cell cycle | *Kif11* | *kinesin family member 11* | 2.5 | 0.007 | 0.843 |
|  | *Ect2* | *ect2 oncogene* | 2.4 | 0.026 | 0.843 |
|  | *S100a6* | *S100 calcium binding protein A6 (calcyclin)* | 2.2 | 0.030 | 0.843 |
|  | *Smc2* | *structural maintenance of chromosomes 2* | 2.1 | 0.004 | 0.843 |
|  | *Snx33* | *sorting nexin 33* | -2.2 | 0.013 | 0.843 |
|  | *Babam1* | *BRISC and BRCA1 A complex member 1* | -2.2 | 0.011 | 0.843 |
|  | *Pttg1* | *pituitary tumor-transforming gene 1* | -2.5 | 0.010 | 0.843 |
| Cell growth/proliferation | *Mki67* | *antigen identified by monoclonal antibody Ki 67* | 2.4 | 0.030 | 0.843 |
|  | *Hgf* | *hepatocyte growth factor* | 2.3 | 0.009 | 0.843 |
|  | *Chchd10* | *coiled-coil-helix-coiled-coil-helix domain containing 10* | -2.1 | 0.038 | 0.843 |
| Cell projection/migration | *Bin2* | *bridging integrator 2* | 2.4 | 0.047 | 0.843 |
|  | *Lims2* | *LIM and senescent cell antigen like domains 2* | -2.8 | 0.034 | 0.843 |
| Chemokine signaling pathway | *Ccl9* | *chemokine (C-C motif) ligand 9* | 3.4 | 0.020 | 0.843 |
|  | *Pf4* | *platelet factor 4* | 3.3 | 0.001 | 0.792 |
|  | *Ccr2* | *chemokine (C-C motif) receptor 2* | 2.6 | 0.002 | 0.843 |
|  | *Ccr5* | *chemokine (C-C motif) receptor 5* | 2.1 | 0.000 | 0.566 |
|  | *Ncf1* | *neutrophil cytosolic factor 1* | 2.1 | 0.002 | 0.843 |
|  | *Pik3r5* | *phosphoinositide-3-kinase, regulatory subunit 5, p101* | 2.0 | 0.021 | 0.843 |
|  | *Rap1b* | *RAS related protein 1b* | 2.0 | 0.011 | 0.843 |
|  | *Adcy2* | *adenylate cyclase 2* | -2.0 | 0.002 | 0.843 |
| Cytoskeleton | *Sdcbp* | *syndecan binding protein* | 2.1 | 0.007 | 0.843 |
|  | *Sgca* | *sarcoglycan, alpha (dystrophin-associated glycoprotein)* | -2.2 | 0.005 | 0.843 |
|  | *Tuba4a* | *tubulin, alpha 4A* | -2.2 | 0.002 | 0.843 |
| Electron transport chain | *Cox6a2* | *cytochrome c oxidase subunit VIa polypeptide 2* | -2.0 | 0.013 | 0.843 |
|  | *Cisd3* | *CDGSH iron sulfur domain 3* | -2.1 | 0.012 | 0.843 |
|  | *Uqcrc1* | *ubiquinol-cytochrome c reductase core protein 1* | -3.5 | 0.005 | 0.843 |
| Extracellular space | *Itga1* | *integrin alpha 1* | -2.1 | 0.014 | 0.843 |
|  | *Mmp12* | *matrix metallopeptidase 12* | 3.0 | 0.031 | 0.843 |
|  | *F13a1* | *coagulation factor XIII, A1 subunit* | 2.7 | 0.003 | 0.843 |
|  | *Lox* | *lysyl oxidase* | 2.5 | 0.006 | 0.843 |
|  | *Sh3bgrl* | *SH3-binding domain glutamic acid-rich protein like* | 2.2 | 0.003 | 0.843 |
|  | *Hspg2* | *perlecan (heparan sulfate proteoglycan 2)* | -2.8 | 0.040 | 0.843 |
| Hydrolase | *Ky* | *kyphoscoliosis peptidase* | -2.4 | 0.037 | 0.843 |
| Immunity | *Lilrb4a* | *leukocyte immunoglobulin-like receptor, subfamily B, member 4A* | 4.6 | 0.005 | 0.843 |
|  | *Cd84* | *CD84 antigen* | 3.5 | 0.002 | 0.843 |
|  | *Lilr4b* | *leukocyte immunoglobulin-like receptor, subfamily B, member 4B* | 2.6 | 0.022 | 0.843 |
|  | *F7* | *coagulation factor VII* | 2.4 | 0.000 | 0.566 |
|  | *Mrc1* | *mannose receptor, C type 1* | 2.3 | 0.000 | 0.575 |
|  | *Adgre1* | *adhesion G protein-coupled receptor E1* | 2.2 | 0.005 | 0.843 |
|  | *Cd300ld* | *CD300 molecule-like family member d* | 2.2 | 0.011 | 0.843 |
|  | *Itgam* | *integrin alpha M* | 2.2 | 0.000 | 0.772 |
|  | *Alcam* | *activated leukocyte cell adhesion molecule* | 2.0 | 0.004 | 0.843 |
|  | *Gm12185; Tgtp1* | *predicted gene 12185; T cell specific GTPase 1* | -2.0 | 0.034 | 0.843 |
|  | *Bst2* | *bone marrow stromal cell antigen 2* | -4.9 | 0.040 | 0.843 |
| Inflammatory response | *Spp1* | *secreted phosphoprotein 1* | 10.0 | 0.001 | 0.843 |
|  | *Anxa1* | *annexin A1* | 3.2 | 0.004 | 0.843 |
|  | *Tlr13* | *toll-like receptor 13* | 2.7 | 0.004 | 0.843 |
|  | *Tlr1* | *toll-like receptor 1* | 2.6 | 0.005 | 0.843 |
|  | *C3ar1* | *complement component 3a receptor 1* | 2.5 | 0.010 | 0.843 |
|  | *Cd180* | *CD180 antigen* | 2.5 | 0.049 | 0.843 |
|  | *Il1rn* | *interleukin 1 receptor antagonist* | 2.2 | 0.027 | 0.843 |
|  | *Havcr2* | *hepatitis A virus cellular receptor 2* | 2.2 | 0.015 | 0.843 |
| Lysosome | *Ctss* | *cathepsin S* | 2.5 | 0.007 | 0.843 |
|  | *Gpr65* | *G-protein coupled receptor 65* | 2.3 | 0.002 | 0.843 |
|  | *Lgmn* | *legumain* | 2.3 | 0.013 | 0.843 |
|  | *Ctsl* | *cathepsin L* | 2.2 | 0.004 | 0.843 |
|  | *Rilpl1* | *Rab interacting lysosomal protein-like 1* | -2.1 | 0.005 | 0.843 |
| Metabolism | *Gstp2* | *glutathione S-transferase, pi 2* | -2.0 | 0.017 | 0.843 |
|  | *Ogdh* | *oxoglutarate (alpha-ketoglutarate) dehydrogenase (lipoamide)* | -2.1 | 0.008 | 0.843 |
|  | *Cs* | *citrate synthase* | -2.4 | 0.018 | 0.843 |
|  | *Pdk2* | *pyruvate dehydrogenase kinase, isoenzyme 2* | -2.9 | 0.013 | 0.843 |
| Metal-binding | *B4galt6* | *UDP-Gal:betaGlcNAc beta 1,4-galactosyltransferase, polypeptide 6* | 2.5 | 0.001 | 0.792 |
|  | *Lcp1* | *lymphocyte cytosolic protein 1* | 2.2 | 0.005 | 0.843 |
|  | *Calr* | *calreticulin* | 2.2 | 0.000 | 0.772 |
|  | *Ablim3* | *actin binding LIM protein family, member 3* | -2.1 | 0.000 | 0.566 |
|  | *Cdh5* | *cadherin 5* | -2.4 | 0.033 | 0.843 |
|  | *Acacb* | *acetyl-Coenzyme A carboxylase beta* | -2.5 | 0.008 | 0.843 |
|  | *Hba-a2; Hba-a1* | *hemoglobin alpha, adult chain 2 (Hba-a2), mRNA.; hemoglobin alpha, adult chain 1* | -2.8 | 0.033 | 0.843 |
| Negative regulation of signal transduction | *Rgs18* | *regulator of G-protein signaling 18* | 2.9 | 0.002 | 0.843 |
|  | *Rgs1* | *regulator of G-protein signaling 1* | 2.1 | 0.007 | 0.843 |
|  | *Rgs5* | *regulator of G-protein signaling 5* | -2.1 | 0.036 | 0.843 |
|  | *Asb15* | *ankyrin repeat and SOCS box-containing 15* | -4.5 | 0.010 | 0.843 |
|  | *Nr1d1* | *nuclear receptor subfamily 1, group D, member 1* | -5.4 | 0.030 | 0.843 |
| Nucleotide-binding | *Hist1h2an* | *histone cluster 1, H2an* | 5.0 | 0.018 | 0.843 |
|  | *Hist1h2af* | *histone cluster 1, H2af* | 4.8 | 0.010 | 0.843 |
|  | *Hist1h2ag; Hist1h2ai* | *histone cluster 1, H2ag; histone cluster 1, H2ai* | 2.7 | 0.025 | 0.843 |
|  | *Hist1h2ai* | *histone cluster 1, H2ai* | 2.6 | 0.016 | 0.843 |
|  | *Hmgb2* | *high mobility group box 2* | 2.2 | 0.044 | 0.843 |
|  | *Hist1h3e* | *histone cluster 1, H3e* | 2.2 | 0.040 | 0.843 |
|  | *Hist1h2ab* | *histone cluster 1, H2ab* | 2.1 | 0.008 | 0.843 |
| Post-transcriptional regulation | *Mir223; F630028O10Rik* | *microRNA 223; RIKEN cDNA F630028O10 gene* | 2.2 | 0.003 | 0.843 |
| Protein synthesis/Translation | *Rpl3* | *ribosomal protein L3* | 2.1 | 0.014 | 0.843 |
| Signal | *Il7r* | *interleukin 7 receptor* | 2.7 | 0.004 | 0.843 |
|  | *Folr2* | *folate receptor 2 (fetal)* | 2.6 | 0.013 | 0.843 |
|  | *Dhrs9* | *dehydrogenase/reductase (SDR family) member 9* | 2.3 | 0.022 | 0.843 |
|  | *Stap1* | *signal transducing adaptor family member 1* | 2.1 | 0.009 | 0.843 |
|  | *Dab2* | *disabled 2, mitogen-responsive phosphoprotein* | 2.0 | 0.005 | 0.843 |
|  | *Perm1* | *PPARGC1 and ESRR induced regulator, muscle 1* | -2.1 | 0.014 | 0.843 |
|  | *Fat4* | *FAT tumor suppressor homolog 4 (Drosophila)* | -2.2 | 0.042 | 0.843 |
|  | *Fmod* | *fibromodulin* | -2.2 | 0.031 | 0.843 |
|  | *Lynx1* | *Ly6/neurotoxin 1* | -2.4 | 0.004 | 0.843 |
|  | *Adgrf5* | *adhesion G protein-coupled receptor F5* | -3.2 | 0.010 | 0.843 |
| Stress response | *Bag3* | *BCL2-associated athanogene 3* | -2.4 | 0.029 | 0.843 |
| Transcriptional Regulation | *Erg* | *avian erythroblastosis virus E-26 (v-ets) oncogene related* | -2.1 | 0.013 | 0.843 |
| Transmembrane | *Msr1* | *macrophage scavenger receptor 1* | 4.3 | 0.001 | 0.843 |
|  | *Cd53* | *CD53 antigen* | 3.2 | 0.008 | 0.843 |
|  | *Ms4a6d* | *membrane-spanning 4-domains, subfamily A, member 6D* | 2.7 | 0.005 | 0.843 |
|  | *Alox5ap* | *arachidonate 5-lipoxygenase activating protein* | 2.6 | 0.001 | 0.843 |
|  | *Atp6ap2* | *ATPase, H+ transporting, lysosomal accessory protein 2* | 2.2 | 0.011 | 0.843 |
|  | *Osbpl8* | *oxysterol binding protein-like 8* | 2.1 | 0.000 | 0.772 |
|  | *Tpd52* | *tumor protein D52* | 2.1 | 0.003 | 0.843 |
|  | *Ms4a6c* | *membrane-spanning 4-domains, subfamily A, member 6C* | 2.1 | 0.001 | 0.843 |
|  | *Ms4a4c* | *membrane-spanning 4-domains, subfamily A, member 4C* | 2.0 | 0.042 | 0.843 |
|  | *Pnpla2* | *patatin-like phospholipase domain containing 2* | -4.4 | 0.005 | 0.843 |
| Unknown | *Hist1h2aj* | *histone cluster 1, H2aj* | 5.3 | 0.010 | 0.843 |
|  | *Gm17482* | *predicted gene, 17482* | 4.4 | 0.037 | 0.843 |
|  | *Gm5593* | *predicted gene 5593* | 2.7 | 0.005 | 0.843 |
|  | *Gm14681; LOC100861774* | *predicted gene 14681 PREDICTED: uncharacterized LOC100861774, miscRNA.* | 2.7 | 0.010 | 0.843 |
|  | *Gm14699* | *predicted gene 14699* | 2.5 | 0.006 | 0.843 |
|  | *Gm13248* | *predicted gene 13248* | 2.4 | 0.009 | 0.843 |
|  | *Gm6291* | *predicted gene 6291* | 2.4 | 0.014 | 0.843 |
|  | *Rpl3-ps2* | *ribosomal protein L3, pseudogene 2* | 2.4 | 0.035 | 0.843 |
|  | *Gm15538* | *predicted gene 15538* | 2.3 | 0.035 | 0.843 |
|  | *Rps12-ps23; LOC100862106* | *ribosomal protein S12, pseudogene 23* | 2.2 | 0.023 | 0.843 |
|  | *Gm10087* | *predicted gene 10087* | 2.1 | 0.001 | 0.843 |
|  | *Rps12-ps21* | *ribosomal protein S12, pseudogene 22* | 2.1 | 0.026 | 0.843 |
|  | *Rps12-ps20* | *ribosomal protein S12, pseudogene 20* | 2.1 | 0.029 | 0.843 |
|  | *Bcl2a1d* | *B cell leukemia/lymphoma 2 related protein A1d* | 2.1 | 0.007 | 0.843 |
|  | *Gm8822* | *predicted gene 8822* | 2.1 | 0.019 | 0.843 |
|  | *LOC100862223* | *PREDICTED: 40S ribosomal protein S12-like* | 2.1 | 0.031 | 0.843 |
|  | *Gm15428* | *predicted pseudogene 15428* | 2.0 | 0.012 | 0.843 |
|  | *Rps12-ps26* | *ribosomal protein S12, pseudogene 26 [Source:MGI Symbol;Acc:MGI:3713332]* | 2.0 | 0.035 | 0.843 |
|  | *Gpd1* | *glycerol-3-phosphate dehydrogenase 1 (soluble)* | -2.0 | 0.007 | 0.843 |
|  | *Klhl33* | *kelch-like 33* | -2.0 | 0.042 | 0.843 |
|  | *Mir3079* | *microRNA 3079* | -2.8 | 0.038 | 0.843 |
| Vesicle | *Atp6v0d2* | *ATPase, H+ transporting, lysosomal V0 subunit D2* | 5.2 | 0.021 | 0.843 |
|  | *Myo5a* | *myosin VA* | 2.0 | 0.000 | 0.566 |
