# Supplementary material for: Cell-type specific effects of mineralocorticoid receptor gene expression suggest intercellular communication regulating fibrosis in skeletal muscle disease
Source: Front Physiol. 2024 Apr 26;15:1322729. doi: 10.3389/fphys.2024.1322729 (PMC11082420; doi:10.3389/fphys.2024.1322729)
Supplement: Supplementary file 3 [file DataSheet1.PDF]

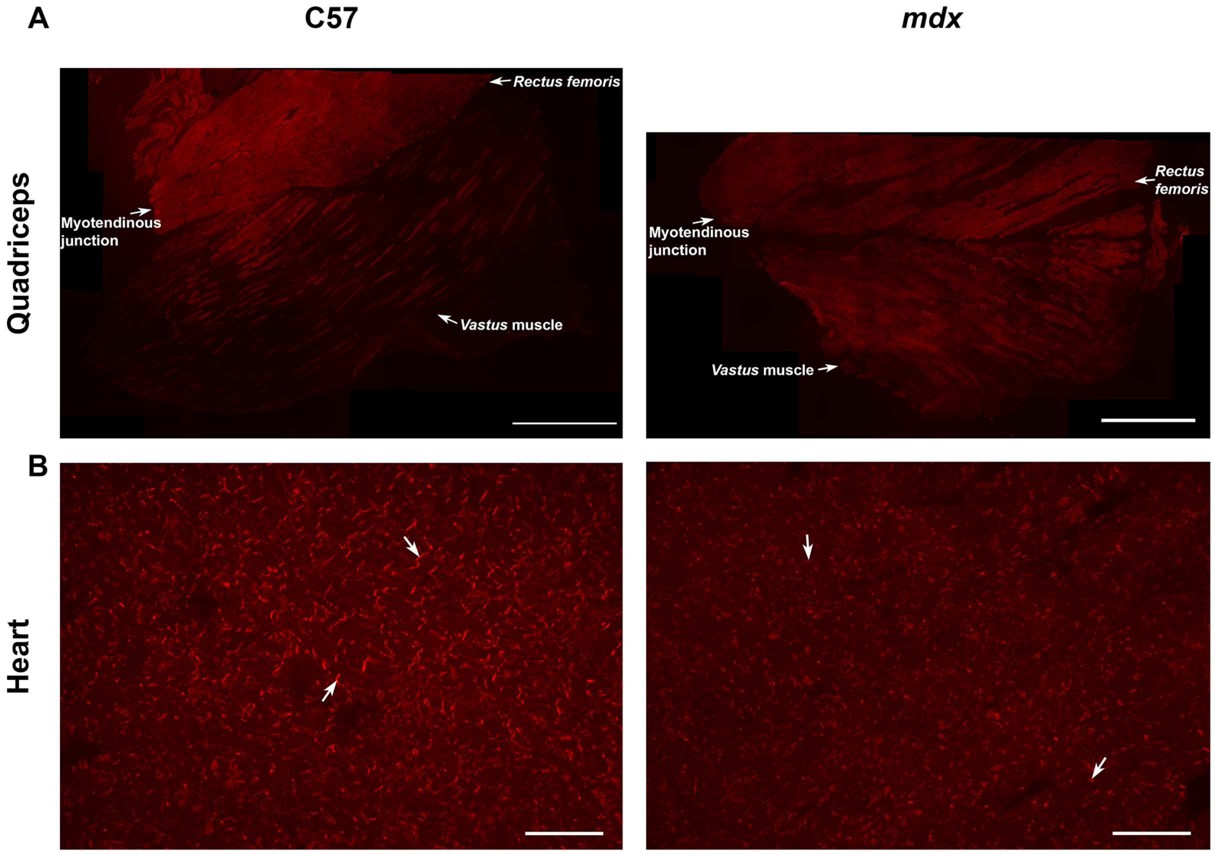

Supplemental Figure 1. Lox localizes uniformly throughout *rectus femoris* in longitudinal sections of quadriceps muscles and to intercalated discs in heart. (**A**) Immunofluorescence staining of Lox in longitudinal quadriceps sections from 8-week-old C57 wild-type and dystrophic *mdx* mice. Bar = 1 mm. The myotendinous junction and the *rectus femoris* and *vastus* heads of the quadriceps are labeled with white arrows and text. (**B**) Immunofluorescence staining of Lox in cross sections of hearts from 8-week-old wild-type C57 and dystrophic *mdx* mice. Examples of intercalated discs are marked by white arrows. Bar = 100 µm.
